# Influence of Partial Disentanglement of Macromolecules on the Rheological, Thermal, and Mechanical Properties of Polypropylene–Polyethylene Blends

**DOI:** 10.3390/molecules30081786

**Published:** 2025-04-16

**Authors:** Justyna Krajenta, Magdalena Lipinska, Andrzej Pawlak

**Affiliations:** 1 Centre of Molecular and Macromolecular Studies, Polish Academy of Sciences, 90-363 Lodz, Poland; justyna.krajenta@cbmm.lodz.pl; 2 Institute of Polymer and Dye Technology, Lodz University of Technology, 90-537 Lodz, Poland; magdalena.lipinska@p.lodz.pl

**Keywords:** polypropylene–polyethylene blends, entanglements, crystallization

## Abstract

The properties of compatibilized blends of polyethylene (PE) and polypropylene (PP), having reduced macromolecular entanglements, were studied. The density of PP macromolecular entanglements was controlled by prior disentangling in solution. The polymer ratio in the blend was 4:1 or 1:4. An ethylene–octene copolymer was used as a compatibilizer. The melt blending process resulted in good dispersion of the minority component, with slightly larger inclusions when more disentangled PP was used. Rheological studies confirmed the achievement of different entanglement densities of PP macromolecules in the blends. The partial disentanglement did not affect the thermal stability of the blends. During the isothermal crystallization studies, faster growth of PP spherulites was observed in the blend with reduced entanglements, which also influenced the entire crystallization process. The recovery time of equilibrium entanglement was investigated and it turned out to be 45 min if the blend was annealed at 190 °C, which was shorter than in the analogous homopolymer. Studies of tensile properties showed that in blends with a majority share of polyethylene, the elongation at break increased with the disentanglement of the minority component, due to better bonding of the blend components and thus the reduction in microcavitation.

## 1. Introduction

The fact that macromolecules are entangled has been known for over 50 years [1]. The presence of entanglements determines many properties of polymers. Examples include rheological properties [2], which affect processing, and mechanical properties, which determine the behavior of polymer products [3]. Also, the crystallization process, which controls the internal structure of the polymer in the solid state, depends on the entanglement of macromolecules [4]. Each polymer has a characteristic equilibrium entanglement density, which can be determined, for example, from its rheological behavior [5]. Several years ago, methods for reducing the entanglement density, which is known as disentangling, were developed. Although it is possible to completely separate macromolecules, it is good for the cohesion of the material to maintain a limited number of entanglements.

Disentangling methods have been developed for homopolymers [6]. The method most commonly used on a laboratory scale is based on the observation that in a dilute solution, macromolecules can be completely separated, and after separation, they have the shape of a coil. When a solid material is required for further studies, a way must be found to transfer the disentangled polymer in solution to the solid state. Typically, the rapid freezing of the solution in liquid nitrogen followed by the sublimation of the solvent is used [7]. If the solution is prepared at a higher temperature and the polymer is able to crystallize during cooling, then crystallization can be used to stabilize the disentangling of the amorphous phase formed from the solution [8]. Another way to stabilize the solution is to add a non-solvent and precipitate the polymer. Other disentangling methods are also being developed that may find industrial application in the future. One of them is crystallization during low-temperature polymerization; the other is the application of complex melt shear during extrusion [9,10].

The properties of disentangled polymers have been the subject of intensive research over the last 10 years. It has been shown that rheological parameters such as storage modulus or zero shear viscosity decrease when the polymer is obtained from a less concentrated solution, i.e., it is less entangled [11]. The mechanical properties, tested in a tensile test, showed a tendency towards a less intense strain-hardening effect in the partially disentangled polymer, which usually favors obtaining a higher deformation [12]. Additionally, a more intensive cavitation process was observed, i.e., the formation of nanovoids and microvoids in the volume of the deformed polymer [13].

The reduction in entanglements influences the crystallization process [10,14,15]. This process was found to occur more rapidly, for example by the faster growth of spherulites. Pawlak et al. [16] showed that changes in crystallization regimes also occur, meaning that better-quality crystals can grow at lower temperatures if the polymer is partially disentangled. It is still controversial whether the glass transition temperature is controlled by entanglement, but recent studies suggest that such dependence exists only when an entanglement density exceeds a critical threshold [17].

Disentangled macromolecules tend to return to an equilibrium state. The re-entanglement is faster when the temperature is higher and the polymer has a lower molecular weight. Knowledge of the re-entanglement time is important for processing the disentangled polymer, also at the laboratory scale. Fortunately, 5–10 min spent by the polymer at a high temperature of 190–200 °C does not cause significant re-entangling [18].

Although the knowledge about disentangled homopolymers is well documented, almost nothing is known about the properties of multicomponent materials formed using the disentangled polymer. Our group was one of the first to conduct research on polymer composites [19,20,21,22]. An interesting observation was the better dispersion of a reinforcing element, such as multi-walled carbon nanotubes, obtained when the macromolecules were more mobile [19]. Much less is known about the properties of polymer blends produced using partially disentangled polymer. Published studies have focused on improving the processability of ultra-high molecular weight polyethylene (UHMWPE) by blending it with lower molecular weight polyethylene [23,24,25,26]. The UHMWPE used was obtained in the polymerization process, which led to a reduction in entanglements [27]. In such a blend made by Tao et al. [25], when the UHMWPE content was 20%, an increase in crystallinity and lamella thickness was observed. In another study by Chen et al. [27], a significant increase in Izod impact strength was observed after using partially disentangled UHMWPE.

As the above review shows, it is necessary to conduct studies on blends of two polymers, including the disentangled one, to find out what effect the partial disentangling of polymer chains has on the formation and properties of the polymer blend. Such blends with partially disentangled macromolecules are the subject of this article. The first one chosen for research was the classical system of two polyolefins: polyethylene and polypropylene.

Blending polymers is a way to modify their original properties and obtain a material with improved, desired properties [28]. One of the earliest blends developed and studied was a blend of polyethylene (PE) and polypropylene (PP), two polymers produced on a large scale. The PP/PE system was treated as a model blend for scientific studies [29,30]. Nowadays, the recycling aspect has also become important because both polymers are among the main components of plastic waste and their complete separation is difficult [31]. The production of the blend is a potential solution to the waste management problem [32].

PP and PE are immiscible at the molecular level and are non-compatible, which leads to the dispersion of the minority component in the form of large inclusions in the matrix, with sizes of 1–50 µm [33], and results in unsatisfactory properties, especially mechanical ones. A solution, which will be discussed later, is the use of a compatibilizer. The morphology of PP–PE blends depends on the proportion of the components, the difference in their viscosity, and the conditions of blending in the melt [34,35,36].

Rheological studies have shown that the viscosity of PP/PE blends is intermediate in relation to the viscosities of the components [37,38]. The observed increase in the size of PE domains dispersed inside PP with the residence time in melt showed that diffusion of PE macromolecules through PP is possible [39]. The thermal stability of PP/HDPE blends was studied by thermogravimetry, and the onset of degradation was found at a temperature intermediate to the temperatures of the components [38].

The mechanical behavior of binary polyolefin blends depends on the composition. Changes in the strength and modulus of elasticity were observed with the change in composition [32,40], although they were not always proportional to these changes and depended on the processing conditions [29,41,42,43]. For example, the tensile strength of PP/HDPE blends was independent of the composition with up to 25% HDPE content [35]. It was also observed that the tensile modulus decreased with increasing PE content when the viscosities of PP and HDPE were similar [35,44]. Measures of ductility such as impact strength or elongation at break always showed negative deviations from additivity [29]. Typically, PP/PE blends show limited elongation at break (i.e., 5–10% of strain), due to the low compatibility of the components [39,45].

In the PP/PE blend, both components can crystallize. Therefore, many authors studied the issue of crystallization. Most of this research focused on polypropylene. No changes in the melting temperature were observed depending on the blend composition [36,46]. The structures growing in the blends with a dominant PP content were spherulites. Their growth rate did not depend on the material composition [39,47,48]. It was observed that PE droplets are surrounded by growing spherulites.

The constant spherulite growth rate means that the changes in crystallization kinetics observed for blends of different compositions are determined by the nucleation. The migration of nuclei from PP to the second component was observed in the case of the blend of PP with low-density polyethylene (LDPE) [49]. The nucleation of PP spherulites in a blend of PP with high-density polyethylene (HDPE) depended on the crystallization temperature. Above 127 °C, nucleation decreased with temperature, while below this temperature, it increased, especially with increasing HDPE content [50]. Similar observations regarding the role of crystallization temperature were made by Eder and Wlochowicz [51], who studied the overall crystallization rate of the PP/LDPE blend. Above 122 °C, crystallization was delayed, while below 122 °C, it was accelerated. Martuscelli et al. [46] and Shanks et al. [52] also observed a delay in the crystallization rate, while Lovinger and Williams [32] observed its acceleration.

There is less information on the crystallization of PE in blends with PP. The melting temperature did not change with the composition [45,46,48]. The dependence of the total crystallization rate on the composition of the blend was observed by Eder and Wlochowicz [51]. When the PP content was 20–50%, the crystallization was delayed, whereas at other contents it was accelerated. The accelerated crystallization of PE in blends was observed by Martuscelli et al. [46,48] and Rybnikar [53]. The enthalpy of crystallization and the heat of the fusion of HDPE and PP in the blend decreased with the increasing amount of the second component [43].

A way to improve the dispersion of the minority component of the blend and increase the adhesion between components, thus obtaining acceptable mechanical properties of the blend, is to add a compatibilizing agent [54,55,56,57]. A compatibilizer is a substance with structural fragments that are miscible or compatible with both polymers. It is also often able to react chemically with one of the polymers. During the blending process, the compatibilizer should reach the interface, increasing the adhesion between the main components of the blend and promoting dispersion [58].

In the case of PE/PP blends, attempts have been made to use block copolymers as compatibilizers [57], such as ethylene–propylene [30,59], ethylene–octene [60], or styrene–ethylene/butylene–styrene [61]. Polyolefin elastomers (e.g., ethylene–propylene–diene elastomer) have also been tested as compatibilizers for blends [62]. In all these cases, the properties of the blends were improved. An option for the compatibilization of PP/PE blends is reactive blending using, for example, polypropylene–grafted maleic anhydride [61] or polyethylene–grafted maleic anhydride [38]. Recently, the use of inexpensive poly(pentadecalactone) as a compatibilizer for PP/HDPE blends has been proposed [63].

In the literature, there are descriptions of the use of other chemical compounds for the compatibilization of polyolefin blends, including non-reactive compatibilizers [64,65]. Although most of the basic research on PP/PE blends was conducted in the 20th century, new research results on these types of blends are still being published [66,67,68,69].

Based on the above knowledge, we focused on the study of blends of partially disentangled PP with entangled HDPE. The properties of these blends have been improved by adding an ethylene–octene compatibilizer (EOC). Since this is one of the first works on blends containing partially disentangled polymers, we decided that an extensive characterization of the examined blends was necessary. The rheology, morphology of the blends, crystallization and crystalline structure, mechanical properties, and thermal stability, which seem to be the most important properties, were investigated.

## 2. Results and Discussion

The experimental procedures are described in the next section, but for ease of discussion of the experimental results, Table 1 lists all analyzed materials and their abbreviations used in the text.

### 2.1. Morphologies of Blends

**Figure 1:**
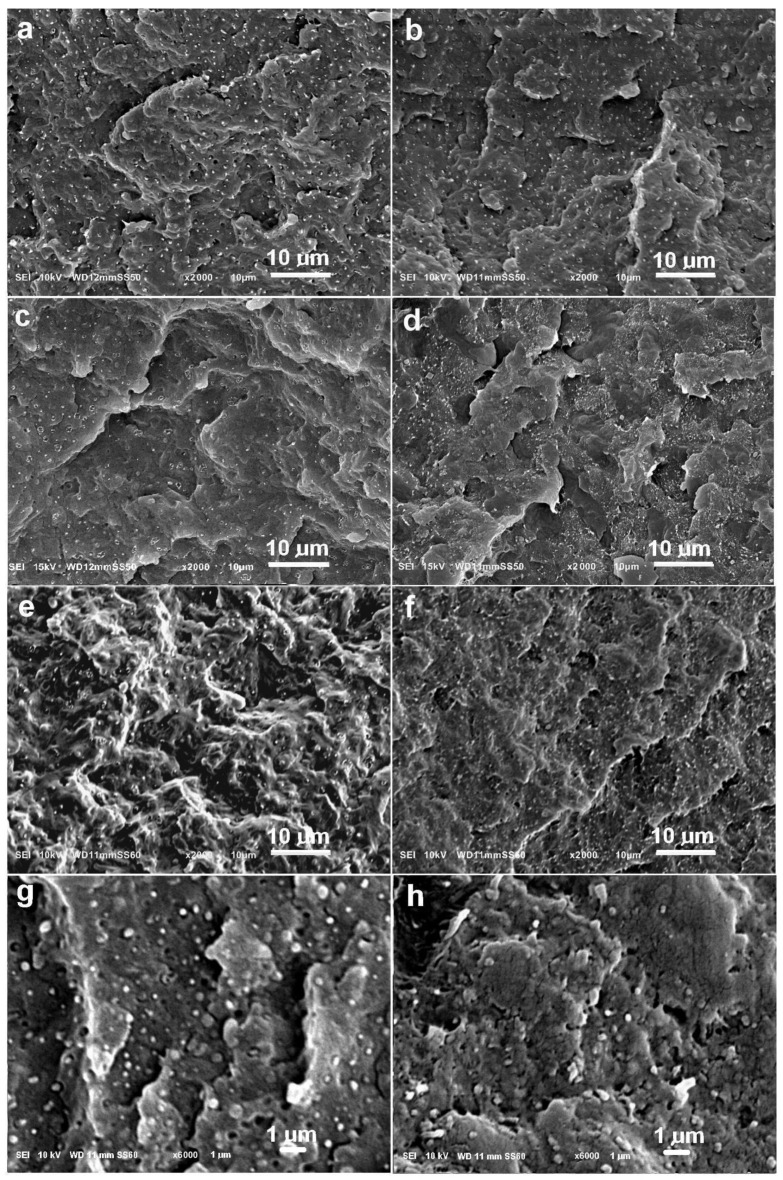
Morphologies of polymer blends: (**a**) PPe/PE (76:19), (**b**) PPe/PE (19:76), (**c**) PP1/PE (76:19), (**d**) PP1/PE (19:76), (**e**) PP05/PE (76:19), (**f**) PP05/PE (19:76). The bars show a size of 10 µm. Higher magnification photos show smaller inclusions of the dispersed phase in the blends: (**g**) PPe/PE (76:19), (**h**) PPe/PE (19:76). The bars here indicate a size of 1 µm.

The morphologies of the prepared blends are presented in Figure 1. All photographs show spherical particles dispersed in the polymer matrix. In the case of blends containing 76 wt.% PP, these are PE inclusions, and in the case of blends containing 76 wt.% PE, these are PP inclusions.

The dispersion of the minority phase in the form of droplets is typical for polyolefin blends [36,41]. In the examined blends, the particles have similar sizes, mostly with a diameter below 1 µm, which indicates that the application of the compatibilizer was effective. Higher magnification photographs were used to measure the detailed sizes of individual particles. Examples of such photographs are shown in Figure 1g,h.

**Figure 2:**
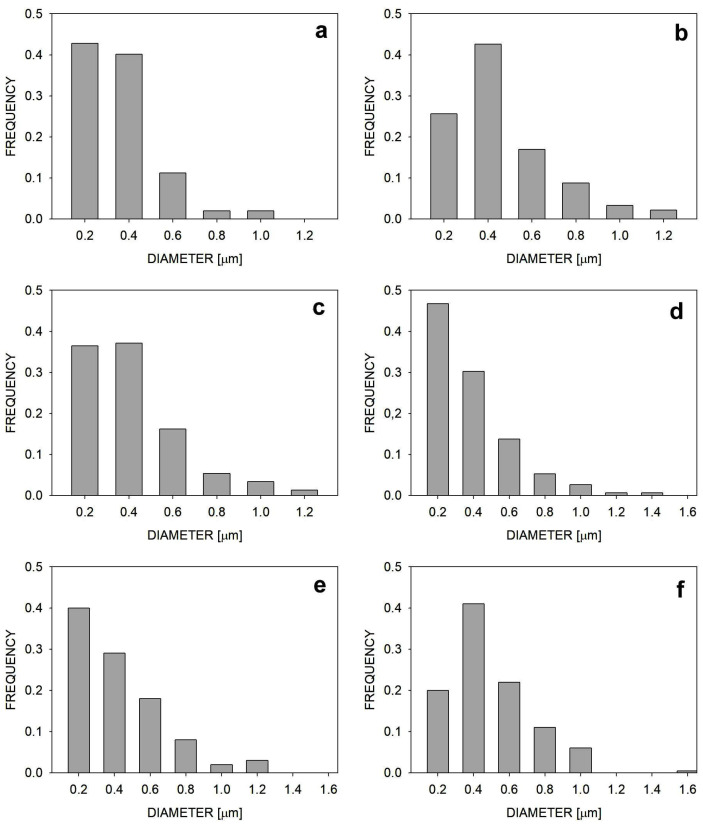
The numerical frequencies of inclusions of dispersed polymer with specific diameters: (**a**) PPe/PE (76:19), (**b**) PP1/PE (76:19), (**c**) PP05/PE (76:19), (**d**) PPe/PE (19:76), (**e**) PP1/PE (19:76), (**f**) PP05/PE (19:76).

Figure 2 shows the frequency of dispersed polymer inclusions as a function of their diameter. Calculations of the average diameter of the dispersed phase for the different blends gave the following results: 0.35 ± 0.18 µm for PPe/PE (76:19), 0.38 ± 0.24 µm for PP1/PE (76:19), 0.44 ± 0.22 µm for PP05/PE (76:19), 0.38 ± 0.24 µm for PPe/PE (19:76), 0.44 ± 0.25 µm for PP1/PE (19:76), and 0.53 ± 0.24 µm for PP05/PE (19:76). This means that the average size of the dispersed phase increased for the blend containing fewer entangled macromolecules, regardless of which polymer was dispersed. The most likely reason for this was the change in the ratio of viscosities of the blended polymers due to the disentangling of one of them, which led to a less effective transfer of forces during blending, causing slightly worse dispersion of the minority component. Analyzing the size distribution, it can be seen that in all blends, there was a small fraction of inclusions larger than 1 µm. Regardless of this, the dispersion of minority components in all blends was very good.

### 2.2. Thermal Stability

**Figure 3:**
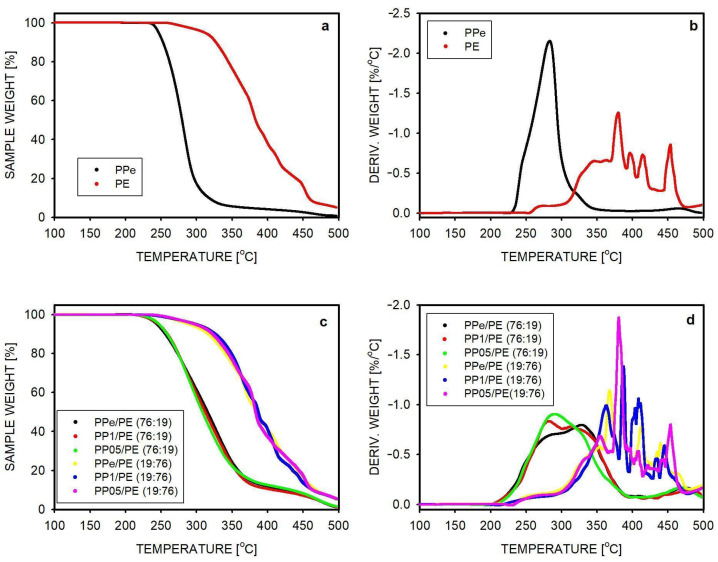
(**a**) The weight loss during heating of homopolymers in airflow as a function of temperature, (**b**) the derivative of the weight change of homopolymers as a function of temperature, (**c**) the weight loss during heating of blends in airflow as a function of temperature, (**d**) the derivative of the weight change of blends as a function of temperature.

The thermal stability of polymers and blends was studied by thermogravimetry. The weight loss as a function of temperature and the derivative of the weight change are shown in Figure 3. The weight changes for PPe and PE homopolymers are shown in Figure 3a,b. PP was thermally stable up to 225 °C, and 50% of weight loss was recorded at T = 280 °C. The residue at 500 °C was 1% of the initial weight. Polyethylene turned out to be a more stable polymer, the degradation of which was observed from a temperature of 257 °C. For this polymer, 50% weight loss was measured at 382 °C, and 5% residue remained at 500 °C. The weight derivative curves show that the degradation process had a single maximum in the case of PPe, while for PE, it was less smooth and characterized by multiple maxima. In the Supplementary Materials (Figure S6), it is shown that the extrusion of homopolymers under similar conditions as for the blends had no effect on the shape of their TGA curves.

The thermal stability of the blends is shown in Figure 3c,d. Degradation effects measured as a function of temperature were similar for blends containing PP with different degrees of entanglement. However, the thermal stability of the blend depended on its composition. The disentangling effect was not significant here because the density of these materials, which controls airflow in the polymer, was independent of the entanglement level. The degradation process started earlier in the blends than in the case of homopolymers. In blends with a PP matrix, the onset of the thermal effect was observed at 200 °C, i.e., 25 °C earlier than in the case of PPe. In the blends with a PE matrix, the onset of thermal destruction occurred at 225 °C, i.e., also at a lower temperature than in the case of PE. The degradation process shown in Figure 3c,d can be divided into two stages, the temperature range of which depends on the composition. For blends containing 76% PP, the first stage takes place at temperatures between 200 °C and 390 °C and mainly involves the degradation of PP, although some participation of PE degradation is probable above 300 °C. PE degradation is certainly visible above 430 °C. When the proportions of the blend components are reversed, i.e., 76% of the blend is PE, the thermogravimetric curves look different. The onset of weight loss, which can be attributed to PP degradation, is observed at 225 °C. At temperatures above 300 °C, PE degradation dominates, with derived weight loss maxima similar to those observed for PE homopolymer.

The TGA results showed that the blends had lower thermal stability than the polymers from which they were made. The degradation process depended on which component, PE or PP, dominated, but the reduction in entanglements had no effect on thermal stability. Figure 3c shows that the degradation effects in the blends start after reaching a temperature of 220–230 °C, i.e., more than 4 min after the sample reached the temperature used during blend preparation (i.e., 180 °C). Since the extrusion time was 3 min, it can be assumed that there was no significant degradation of the material occurring as a result of the blending process.

### 2.3. Rheology

**Figure 4:**
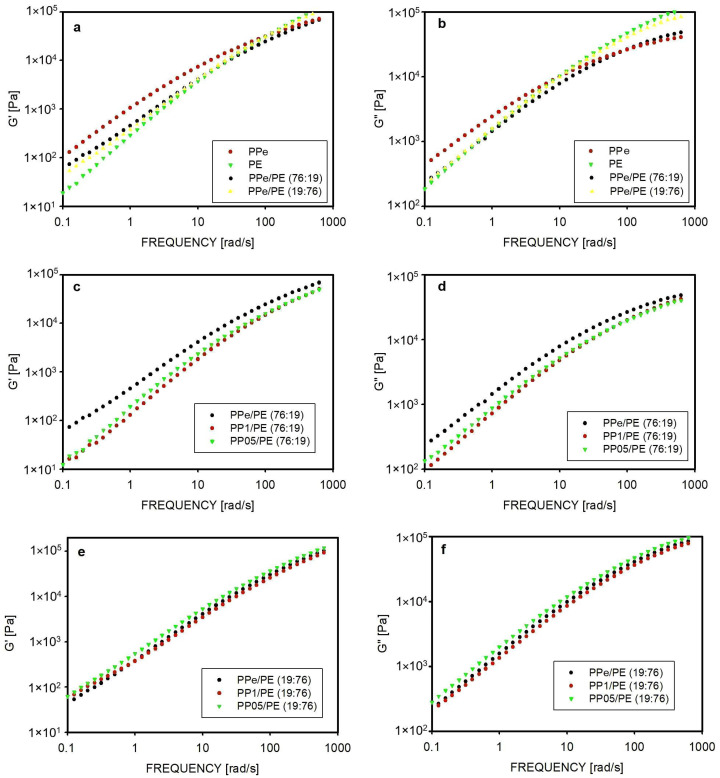
The rheological properties measured by a frequency sweep test: (**a**) storage modulus of homopolymers and entangled blends, (**b**) loss modulus of homopolymers and entangled blends, (**c**) storage modulus of 76:19:5 wt.% blends, (**d**) loss modulus of 76:19:5 wt.% blends, (**e**) storage modulus of 19:76:5 wt.% blends, (**f**) loss modulus of 19:76:5 wt.% blends.

The rheological properties of the polymers and blends were examined using a frequency sweep test. The results are shown in Figure 4. The dependence of the storage modulus G′ and loss modulus G″ on frequency for PPe and PE homopolymers is presented in Figure 4a and Figure 4b, respectively. At low frequencies, the moduli for PPe are higher, while at high frequencies, the moduli measured for PE are higher. The G′ and G″ values for the blends depend on the properties of both components, although the majority component has a greater influence. For this reason, at low frequencies, the G′ and G″ values for the PPe/PE blend (76:19) were much lower than the levels for PPe. However, from a frequency of 100 rad/s, the influence of the presence of PE was more visible and the G′ modulus for this blend was close to G′ for PPe, and the G″ modulus for the blend was even higher than for PPe. When PE was the main component of the blend, as in the case of PPe/PE (19:76), the moduli at low frequencies were more similar to those of PE than to those of PP. At higher frequencies, the values of the moduli of the blends were similar, while in the frequency range of 100–512 rad/s, the moduli G′ and G″ were higher for the PPe/PE blend (19:76) than for the PPe/PE blend (76:19). For example, at a frequency of 400 rad/s, the storage modulus values were 86,300 Pa for PE, 77,600 Pa for the PPe/PE (19:76) blend, 54,300 Pa for the PPe/PE (76:19) blend, and 59,400 Pa for PP.

Figure 4c,d show the rheological properties of the blends in which the matrix was PP and the density of PP entanglements was reduced. In the whole experimental range, the G′ and G″ moduli for PP1/PE (76:19) and PP05/PE (76:19) had lower values than for the blend with equilibrium entangled PP. This is due to the easier flow during the shearing of less entangled macromolecules. In the low-frequency range, the lowest values of G′ and G″ moduli were observed for the PP1/PE (76:19) blend, while in the high-frequency range, the relationship between the moduli of the disentangled blends was inverse and the lowest moduli were observed for the PP05/PE (76:19) blend. Since the high-frequency range is taken into account when determining the level of the entanglement of macromolecules, the G′ moduli values of the blends at these frequencies confirmed that the macromolecules in the PPe/PE (76:19) blend were the most entangled and were the least entangled in the PP05/PE (76:19) blend.

The effect of disentanglement on the rheological properties should be much less visible for those blends where PP is the minority phase. The results of the frequency sweep test for PPe/PE (19:76), PP1/PE (19:76), and PP05/PE (19:76) blends are shown in Figure 4e,f. For these blends, the sets of dots representing the G′ and G″ moduli are located close to each other.

The dispersed phase inclusions in the blends, as shown in the SEM photographs, had typical dimensions below 1 µm, and at these sizes, even the easier movement of disentangled polypropylene macromolecules inside the inclusion had no influence on the rheological behavior of the dominant polyethylene fraction.

### 2.4. Isothermal Crystallization

It is known from studies of homopolymers that the reduction in entanglements influences crystallization, among others, by increasing the overall crystallization rate [3,4,6,16]. The overall crystallization rate depends on the nucleation density and the crystal growth rate [16,18,20]. We performed crystallization studies under isothermal conditions using the DSC technique and then observed the growth of spherulites on a hot stage connected to a polarizing microscope. The temperature profile of the DSC tests was selected in a such way that the sample first melted (at 190 °C), then, after cooling to 135 °C or 137 °C, PP crystallization occurred, and after its completion and lowering the temperature, the isothermal crystallization of PE began at 123 °C or 125 °C.

An example DSC thermogram showing such an experiment is included in the Supporting Materials, Figure S10. The choice of two crystallization temperatures for each polymer was intended to help show possible differences related to the kinetics of the crystallization process at different temperatures. Since the EOC compatibilizer had a low melting point (47 °C), it remained a liquid under the conditions at which crystallization was performed.

**Figure 5:**
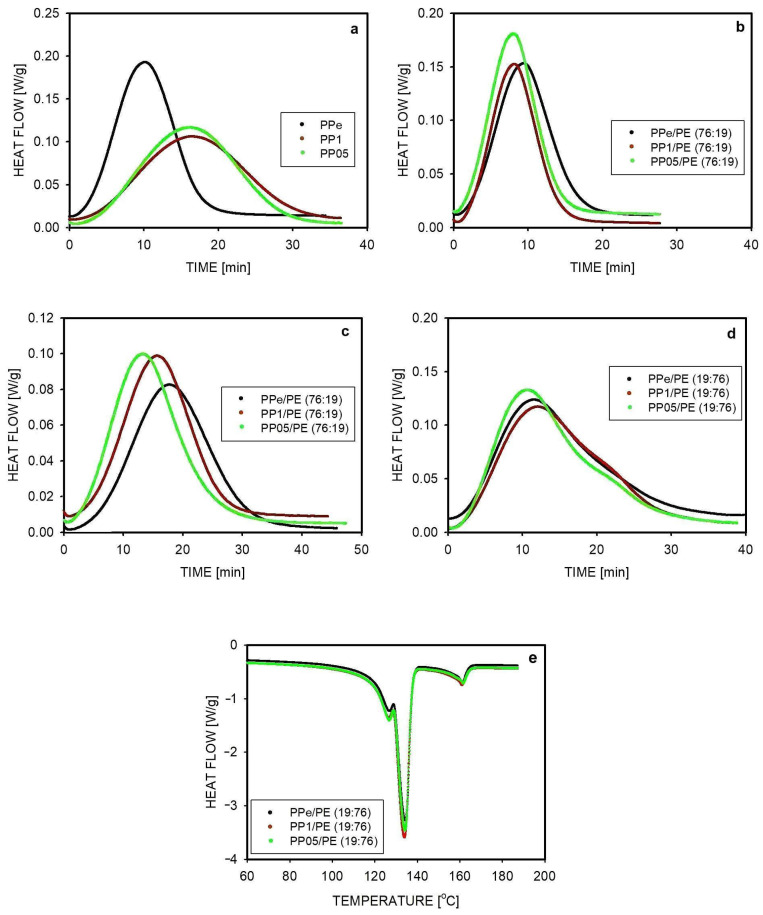
The heat flow during isothermal crystallization: (**a**) the crystallization of differently entangled PP at 135 °C; (**b**) the crystallization of PP in blends in which its content was 76 wt.%, carried out at a temperature of 135 °C; (**c**) the crystallization of PP in 76:19 blends at 137 °C; (**d**) the crystallization in blends with a composition of 19:76 carried out at a temperature of 125 °C; (**e**) melting samples of blends with a composition of 19:76 after crystallization at 125 °C.

The course of isothermal crystallization of PP samples with different entanglement densities is shown in Figure 5a as a dependence of heat flow on time. In all cases, the crystallization process started soon after reaching the selected temperature. The conversion of the melt into the solid state was the fastest in the case of PPe. It took 23 min, and the maximum heat flow was recorded after 10 min. The crystallization of two disentangled PPs proceeded similarly to each other but was slower than in the case of PPe. The maximum heat flow was recorded after 16 min, and the process was completed after 35 min. The reason for the slower crystallization in PP1 and PP05 was the lower nucleation than in PPe, dominating the contribution from the higher crystal growth rate in the disentangled PPs. Previous studies have shown that some nuclei are removed during the disentangling of the polymer in solution [16].

Interesting were the observations of the isothermal crystallization of PP in blends in which it was the main component, i.e., PPe/PE (76:19), PP1/PE (76:19), and PP05/PE (76:19) (Figure 5b). The crystallization time of the entangled PP in the blend was similar to that for PPe, e.g., the maximum heat flow was reached after 10 min. Since the crystal growth rate of PPe is slower in the blend, as will be discussed later, similar crystallization times indicate increased nucleation of PP. This must be the result of the activation of additional nuclei due to the blend formation. Additional nuclei were also formed in blends containing partially disentangled PP, which furthermore had a higher crystal growth rate of PP compared to PPe. The growth rates will be discussed later. As a result of these factors, the crystallization time of the disentangled PP1 and PP05 in the blends, which was 18 and 17 min, respectively, was much shorter than that of homopolymers (35 and 33 min, respectively) and shorter than the crystallization time of PPe in the PPe/PE (76:19) blend, which was 22 min (Figure 5b).

The tendency for faster crystallization in the blend of less entangled PP is even more evident when the time of crystallization is longer, as in the case of crystallization at 137 °C. Then, at a higher temperature, fewer nuclei are active and the differences in growth rates have a greater impact on the entire crystallization process. This is illustrated in Figure 5c, where it can be seen that the PP present in the most disentangled blend, i.e., PP05/PE (76:19), crystallized the fastest. The maximum heat flow was observed after 13 min for the PP05/PE (76:19) blend, 15 min for PP1/PE (76:19), and 18 min for the PPe/PE (76:19) blend.

The heats of crystallization and degrees of crystallinity for the polypropylenes and blends shown in Figure 5a,b were determined and are presented in Table 2. The crystallinity of PP as a homopolymer and in the blends crystallized at 135 °C was similar and was in the range of 46–50%. Also, when crystallization took place at 137 °C, a similar degree of PP crystallinity was achieved in homopolymers and blends (45 to 49%). This means that if sufficient time was given for crystallization, it occurred completely, at a level typical for this polymer.

The crystallization of PP was also studied when PP was dispersed in the PE matrix. Regardless of the degree of PP disentanglement, the crystallization in the droplets of the dispersed phase proceeded differently than in the matrix. First, it was observed that PP was unable to start crystallization within 40 min of the experiment if the temperature of crystallization was 127 °C or higher. In small drops, the number of active nuclei was limited, especially at a higher crystallization temperature, and what is more, as is known from the literature, the migration of nuclei from the PP phase to the PE matrix could occur [50]. Only after lowering the temperature to 125 °C, crystallization was observed in these blends (see Figure 5d). However, this is the temperature at which polyethylene can also crystallize. The time dependences of the heat flow for blends with differently disentangled PP look similar in Figure 5d.

The heat of crystallization was 167 J/g for PPe/PE (19:76), 155 J/g for PP1/PE (19:76), and 154 J/g for PP05/PE (19:76). The high values of the measured heats of crystallization, taking into account that the share of PP was 19%, confirmed that both PP and PE crystallized at 125 °C. Their contributions to the total crystallinity can be estimated from the heat of the fusion of samples heated immediately after crystallization. Figure 5e shows the melting of previously crystallized samples. The results of the total heat of fusion measurements showed that it was consistent with the previously determined heat of crystallization, and the PP contribution to this heat was 13.2–14.0 J/g. Taking into account the share of PP in the total mass of the sample, this corresponds to a degree of crystallinity of 35% for PPe/PE (19:76), the same for PP1/PE (19:76) and 33% for PP05/PE (19:76). Calculations of the degree of crystallinity for PE gave values of 64% for PPe/PE (19:76), 70% for PP1/PE (19:76), and 69% for PP05/PE (19:76).

**Figure 6:**
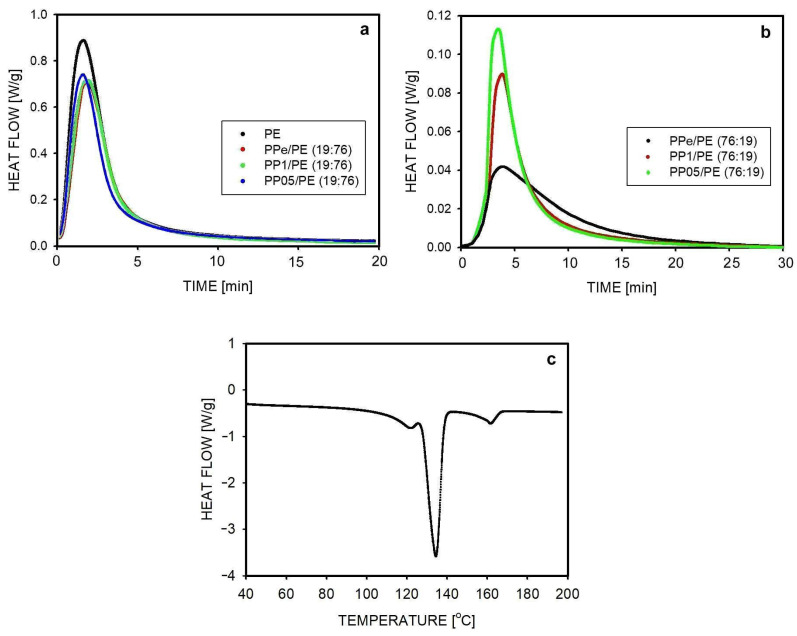
The heat flow during isothermal crystallization: (**a**) the crystallization of PE in blends of composition 19:76 at 123 °C. The red curve almost overlaps the green curve. (**b**) The crystallization of PE in blends of composition 76:19 at 123 °C. (**c**) The melting of the PP1/PE (19:76) blend after isothermal crystallization at 123 °C.

The crystallization of PE in the blend was studied in more detail using the example of isothermal crystallization at a temperature of 123 °C, carried out in blends in which PE was the main component. The results of heat flow measurements as a function of time are shown in Figure 6a,b. The crystallization began just after reaching the desired temperature. The heat flow curves for PPe/PE (19:76), PP1/PE (19:76), and PP05/PE (19:76) look similar and are close to the curve representing the crystallization of PE, also shown in Figure 6a. This means that the degree of the disentanglement of macromolecules in PP droplets did not affect the crystallization of the matrix. According to Figure 6a, the measured heat flow increased quickly, reaching the maximum value after about 2 min. The crystallization of PE at 123 °C was completed after approximately 15 min, which is much shorter than during the previously discussed crystallization at 125 °C. PP crystals could also have been formed during this crystallization, but it can be assumed that this occurred during cooling, before reaching the temperature of 123 °C. Measurements of the areas under the curves provided information on crystallinity, and these results are presented in Table 2. An increase in crystallinity was observed from 64% for PE to 72% for blends, similar to all blends with a PE matrix.

To ensure that PP crystallization did not contribute to these results, the samples were melted after crystallization and the melting process was analyzed. For example, the melting of the PP1/PE (19:76) sample is shown in Figure 6c. During melting, two main peaks were observed, one with a maximum at 134 °C, representing the melting of PE, and the other at 162 °C representing the melting of PP. The heat of fusion was 209 J/g for PE and 100 J/g for PP. The first result agrees with the result obtained from the crystallinity measurement (Table 2 and Figure 6a), which means that PP indeed crystallized during cooling, before reaching the temperature T = 123 °C. The increased degree of PE crystallinity in these blends, compared to the similarly crystallized homopolymer (72% vs. 64%), may be due to some migration of nuclei from PP to PE.

An analysis of the crystallization of PE dispersed in PP was also performed. The crystallization process of PE was studied at a temperature of 123 °C, so it occurred after the crystallization of PP at 133 °C. The raw DSC thermograph illustrating the applied procedure is shown in Figure S10 (see Supplementary Materials). The heat flow as a function of time during the crystallization of PE in the three studied blends is shown in Figure 6b. The crystallization started shortly after reaching the temperature of 123 °C. The maximum heat flow was recorded after 3–4 min, slightly earlier when PE was dispersed in the less entangled PP. Crystallization was completed after about 25 min, similar to all studied blends. The shape of the heat flow curve as a function of time was different for PPe/PE (76:19) than for both blends produced using disentangled PP. The differences concerned the areas under the curves, and thus the final crystallinity of the blends. In all blends with a composition of 76:19, the crystallinity of PE was lower than in the homopolymer (36–43% vs. 64%). This is probably due to the reduction in activity and the number of nuclei caused by the extrusion process. The known process of nuclei migration from PP to PE during long-term isothermal crystallization differentiated the blends [49]. Since the highest crystallinity of PE in the blend was obtained when PP was less entangled, this is probably due to easier migration of the nuclei from the matrix volume through the less entangled chains of PP.

**Figure 7:**
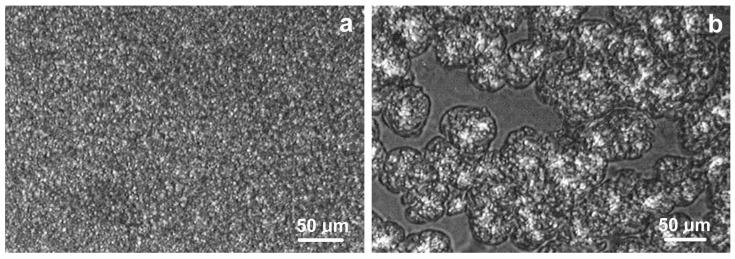
(**a**) Morphology of PP05/PE (19:76) blend after crystallization at 123 °C, observed in a polarized optical microscope. A fine crystal structure is visible; (**b**) Spherulites of PP growing in the PP05/PE (76:19) blend at 135 °C. The polarizers in this case were not completely crossed in order to better show the boundaries of spherulites.

The crystallization of the blend was also examined in thin films using polarized optical microscopy. Of course, it was not possible to study the process inside inclusions smaller than 1 µm, but it was possible to observe the crystallization of the bulk polymer surrounding the inclusions. The observations carried out showed that when the blend contained 76 wt.% PE, the crystal structures growing at temperatures of 123–125 °C were too small for further analysis (Figure 7a). However, when the main component of the blend was polypropylene and the crystallization took place at 133–135 °C, spherulitic structures with dimensions of 50–100 µm were visible. They can be used to measure the crystallization rate and to estimate the density of spherulitic nucleation. Figure 7b shows spherulites growing at 135 °C in the PP05/PE (76:19) blend. At the time the photograph was taken, the crystallization process had not yet been completed.

Since the radial growth direction of PP spherulites is the same as the growth direction of lamellae, the crystal growth rate can be calculated by measuring the change in the position of the spherulite boundary as a function of time. It has been previously shown that the crystal (spherulite) growth rate increases when the density of macromolecular entanglements is lower [4,70], so this can be considered as an additional confirmation of the achievement of macromolecular disentanglement. The photograph in Figure 7b shows that most of the spherulites are of similar size, indicating simultaneous nucleation on heterogeneous nuclei. In all PP-rich blends, spherulite growth started immediately after reaching the target temperature.

The results of the measurement of the growth rate of PP spherulites in differently entangled homopolymers and in blends with a dominant share of PP are shown in Table 3. When the crystallization was performed at 135 °C, the growth rate of spherulites in the homopolymer depended on the degree of entanglement, increasing from 3.90 µm/min for equilibrium entangled PPe to 5.40 µm/min for partially disentangled PP05.

The reason for this increase is primarily the easier transport of macromolecules by diffusion to the crystal growth surface. This is consistent with the crystallization theory [71,72], which requires that a sufficiently long fragment of a macromolecule being incorporated into a crystal should not be limited by entanglements with other macromolecules. Therefore, reduced polymer entanglement has a positive effect on the transport of the macromolecule through the melt and its incorporation into the resulting crystal.

The growth rates of spherulites in blends with a dominant PP fraction were different than in the homopolymer. The well-dispersed PE particles were obstacles to the propagation of spherulites, so the growth rate in these blends was reduced, for example from 3.90 µm/min for fully entangled PPe to 3.43 µm/min for the PPe/PE (76:19) blend. When less entangled PP was used, the growth rate of spherulites in the blend increased. The ratio of the spherulite growth rate in the blend to the spherulite growth rate in the homopolymer was close to 0.9 in all three cases.

Table 3 also contains the results of the spherulite nucleation density measurement. The influence of macromolecular entanglement on nucleation is only beginning to be studied. Yamazaki et al. [73] suggested that the state of disentangling should favor nucleation. However, this applies rather to homogeneous nucleation and may not be observed in the case of heterogeneous nucleation, which occurs in our blends.

In the case of nucleation in PP with different degrees of macromolecular entanglement, it can be seen that there were more nuclei in PPe than in both disentangled polymers (i.e., PP1, PP05). This was probably due to the disentangling of PP in solution, during which some of the nuclei were removed [16]. Significantly more PP spherulites were nucleated in the blends with the PP matrix than in the homopolymers. The increase was 62% for PPe/PE (76:19), 49% for PP1/PE (76:19), and 28% for PP05/PE (76:19), in all cases relative to the corresponding homopolymer. The most probable reason was the facilitated nucleation at the polymer interface. The smaller increase in nucleation density in the more disentangled PP could be due to the easier migration of nuclei into the PE inclusions.

**Figure 8:**
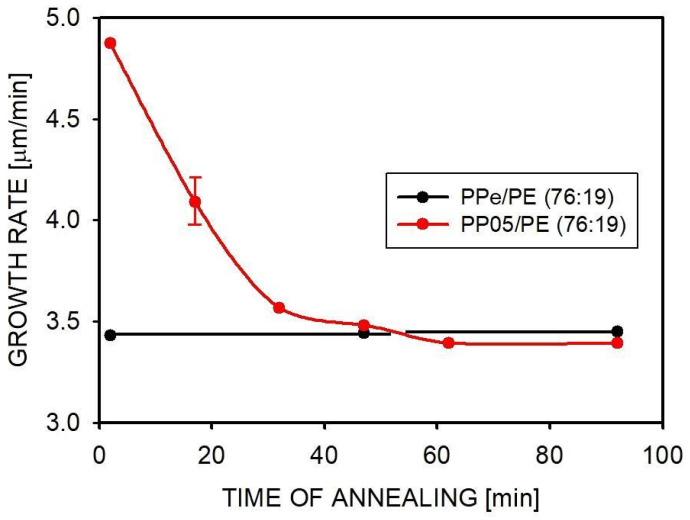
Growth rate of PP spherulites, measured at 135 °C as a function of the annealing time of the blends at 190 °C.

When studying disentangled polymers, the kinetics of their re-entanglement is important. We have previously performed similar studies for the currently examined PP homopolymer and found that the re-entanglement process is not very fast, which provides an opportunity to measure the properties before the polymer re-entangles [18]. Since there was no similar information available for blends in general, we measured the spherulite growth rate after different annealing times of the PP05/PE (76:19) blend at 190 °C. The results are shown in Figure 8.

The spherulites in the blend with initially limited entanglements grew slower with increasing annealing time. After 45 min, the growth rate decreased from 4.9 to 3.4 µm/min, i.e., to the rate measured for PPe/PE (76:19), and remained constant after 60 min. No change in the growth rate of PPe/PE (76:19) spherulites was observed during annealing, which further indicated that the experiment was not influenced by the possible degradation of polymers. In previous studies conducted for PP05 and PPe, it was observed that the spherulite growth rate decreased to the rate corresponding to the equilibrium state after 70 min when the polymer was annealed at 200 °C and after 90 min when annealing was carried out at 185 °C [18]. Referring to these results, it can be stated that the entanglement of macromolecules occurs faster in the blend. A similar reduction in entanglement time of PP compared to homopolymer was observed in the PP/Al_2_O_3_ nanocomposite [20]. It can be assumed that the faster entanglement of macromolecules in the blends is due to the presence of the dispersed phase, but this issue requires further research. In conclusion, short 2–3 min processing at a high temperature has only a small effect on entanglement macromolecules in the blend.

### 2.5. Mechanical Properties

**Figure 9:**
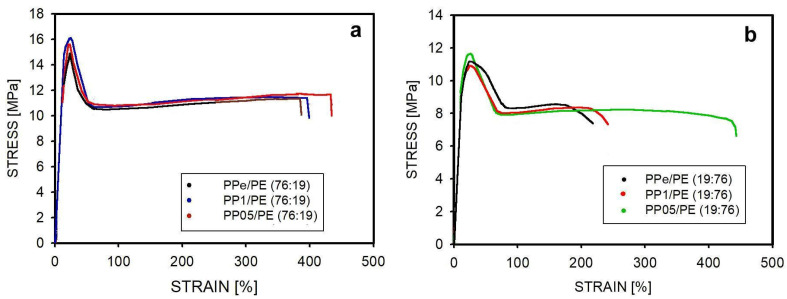
Typical stress–strain relationships for the tested blends: (**a**) blends containing 76 wt.% PP; (**b**) blends containing 19 wt.% PP.

It is known that PP and PE blends, even after compatibilization, are brittle and break quickly if deformed at ambient temperature [39,45]. Therefore, studies on the effect of macromolecule disentanglement on mechanical properties are better performed at higher temperatures, where it is possible to obtain a higher elongation at break. Our mechanical experiment was carried out at a temperature of 60 °C. The results of the tensile test are shown in Figure 9 and Table 4.

Figure 9 shows selected stress—strain curves for samples that reached large deformations. The strain values for the samples shown in this figure are higher than the averages given in Table 4, indicating that many of the tested blends contained structural defects that caused the material to break more quickly. These defects were probably single larger agglomerates or impurities. The curves in Figure 9 illustrate better than the data in Table 4 the potential deformation properties of the materials and the fact that there was no strain-hardening phase.

All tested blends were capable of significant deformation, beyond the yield point. The yield stress was 15–16 MPa for blends with a high PP content and about 11 MPa for blends with a high PE content. The slightly higher values noted for blends with a partially disentangled polymer could be due to better bonding of the components at the interface, perhaps due to easier penetration of this interface by less entangled macromolecules. However, the effect was relatively weak. There were no significant differences in the yield strain between blends with the same PP content.

At the yield point, plastic deformation was initiated with the formation of a neck in the tested sample. This is visible on the experimental curve (Figure 9) as a decrease in the measured force. At the experimental temperature, the neck propagated through the specimen, with the appearance of a plateau in the measured stress. Due to the relatively high temperature of the experiment, no classical strain hardening was observed [74], but after reaching a certain stress level (11 MPa for PP-rich blends and 8 MPa for PE-rich blends), the sample fractured. The presence of soft inclusions of dispersed PE in the harder PP, even if it was less entangled, caused the influence of different degrees of matrix entanglement on the tensile properties to be invisible.

A different situation occurred when the harder, differently entangled PP was dispersed inside the softer PE. In this case, when comparing the blends PPe/PE (19:76), PP1/PE (19:76), and PP05/PE (19:76), a relationship was observed between the average strain at break and the degree of the entanglement of macromolecules. It is difficult to expect that spherical PP inclusions would undergo significant and different deformations, even if they were less entangled, but it is possible that easier penetration of PE macromolecules into the less entangled PP would provide better cohesion of both polymers and thus a greater deformability.

**Figure 10:**
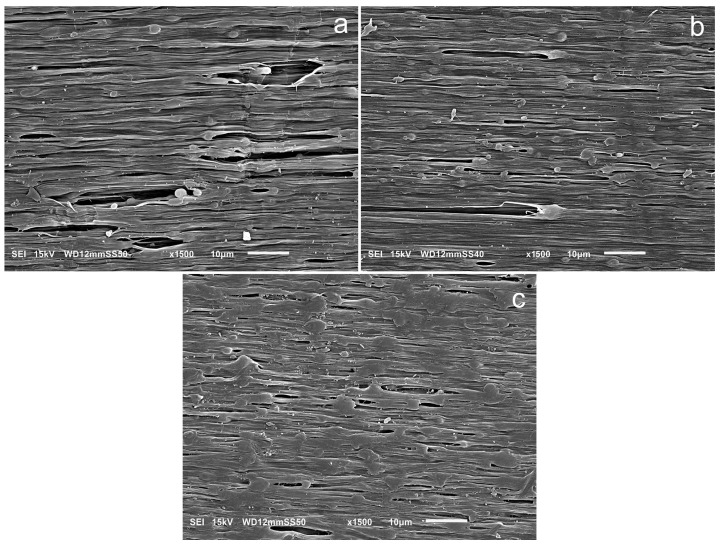
Morphologies of highly deformed (i.e., shortly before breaking) samples of (**a**) PPe/PE (19:76), (**b**) PP1/PE (19:76), and (**c**) PP05/PE (19:76).

To understand the reasons for the differences in elongation at break, microscopic observations of the samples after deformation were performed. We focused especially on the most deformed parts of the samples. SEM images of the entangled PPe/PE (19:76) blend and the partially disentangled PP1/PE (19:76) and PP05/PE (19:76) blends are shown in Figure 10. The images show the surfaces of the most deformed parts of the samples, close to the fracture site, but not including it. The direction of deformation was horizontal.

Figure 10a shows that in the blend containing fully entangled PP, many large voids of 20–40 µm in length and 2–5 µm in diameter, oriented in the deformation direction, were formed as a result of stretching (Figure 10a). Many of these voids have spherical inclusions visible at their ends. Their position inside the voids indicates that cavitation was initiated there due to stress inhomogeneity. Only single such voids are visible in the image showing the PP1/PE (19:76) blend (Figure 10b). Significantly fewer holes are visible in the image of the deformed PP05/PE (19:76) sample (Figure 10c), even though this sample was almost twice as elongated as the PPe/PE (19:76) sample and could be expected to have more and larger voids. Moreover, the photographs in Figure 10 show that the dispersed phase inclusions were less separated from the PE matrix if the PP used was less entangled. The better bonding of the blend components, limiting the cavitation, allowed for obtaining higher elongations at break, which explains the results in Figure 9b.

**Figure 11:**
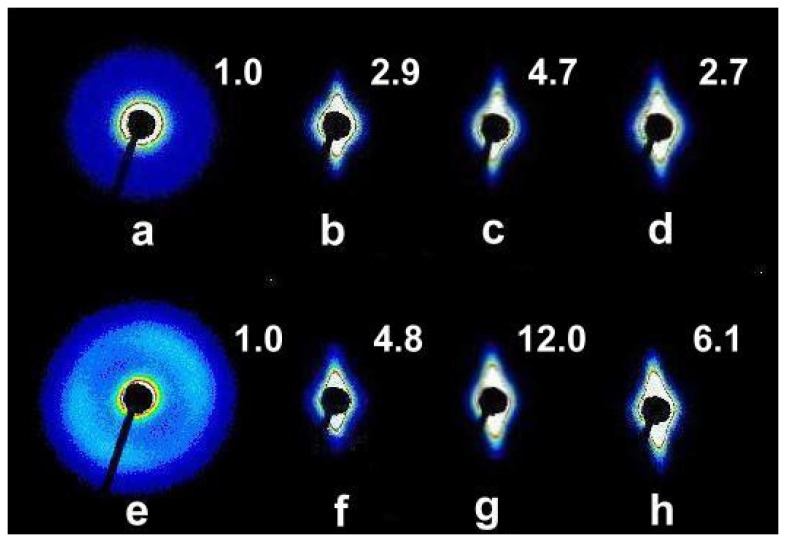
SAXS patterns recorded for the blends studied: (**a**) PPe/PE (76:19) before deformation, (**b**) PPe/PE (76:19) after deformation, (**c**) PP1/PE (76:19) after deformation, (**d**) PP05/PE (76:19) after deformation, (**e**) PPe/PE (19:76) before deformation, (**f**) PPe/PE (19:76) after deformation, (**g**) PP1/PE (19:76) after deformation, (**h**) PP05/PE (19:76) after deformation. The numbers near the scattering images show the ratio of the scattering intensity to the scattering intensity from the non-deformed, equilibrium entangled blend. Deformation direction was horizontal.

In order to obtain a more complete picture of the morphological changes in the blends as a result of deformation, X-ray studies using the SAXS technique were also performed. In materials capable of forming pores under the influence of deformation, one would expect that in addition to microvoids, there would also be nanovoids, which are detectable by SAXS. The scattering images recorded before and after deformation are shown in Figure 11. The upper part of Figure 11 shows the scattering for blends with a high PP content, and the lower part shows the scattering for blends with a dominant PE content. The positions of the places where measurements were made in the deformed samples correspond to those in Figure 10.

The scattering patterns show that no significant orientation existed in PPe/PE (76:19), PPe/PE (19:76), and other blends prior to deformation. The low intensity of the signals shows that this scattering originated from periodic lamellar structures. Due to the overlapping scattering on the PP and PE crystals, their sizes were not determined, also because it was expected that during deformation, the signal from the lamellae would be suppressed by the stronger signal coming from the nanovoids formed during deformation.

The scattering patterns from the deformed blends show a strong orientation and an increase in scattering intensity. The numbers next to the scattering patterns in Figure 11 show the ratio of the total scattering intensity for each of the deformed samples to the total scattering intensity for the non-deformed sample. The significant increase in scattering intensity on the deformed samples indicates that it comes from the formed nanoholes and not, for example, from an increased number of crystals. The elongated shape of the scattering pattern indicates that the formed nanocavities were ellipsoidal and oriented in the direction of deformation.

It is characteristic that the scattering is strongest on voids created in blends with a dominant share of PE. In the case of the PP1/PE (19:76) blend, it is as much as 12 times stronger than on the non-deformed PPe/PE (19:76) blend. In blends with a dominant share of PP, the most intense scattering also occurred when the PP was slightly disentangled, i.e., PP1 was used, but for these blends, the measured intensities and differences were smaller than for blends with a high PE content. In the examined blends, no simple dependence of the scattering intensity on the degree of macromolecule disentanglement was observed, which was previously demonstrated for the PP homopolymer [13]. The issue of structural changes at the micro level occurring during the plastic deformation of blends and determining macroscopic properties will be the subject of future research.

## 3. Materials and Methods

### 3.1. Materials

Isotactic PP Novolen 1100 N (BASF, Ludwigshafen, Germany) with a molecular mass of 250 kg/mol, molecular mass distribution of 5.0, density of 0.936 g/cm^3^, and melt flow index of 11 g/10 min (2.16 kg, at 230 °C) was used to prepare disentangled samples. Hostalen GC 7260, a high-density polyethylene with a density ρ = 0.960 g/cm^3^ and a melt flow index of 8 g/10 min (at 190 °C, 2.16 kg, according to ISO 1133 [75]) was supplied by Lyondell Basell (Houston, TX, USA). To ensure the compatibility of the blend components, Engage 8180 ethylene–octene copolymer (polyolefin elastomer) (EOC) from Dow Chemical Company (Midland, TX, USA) was used. According to the manufacturer, its density is 0.863 g/cm^3^, its melt flow index is 0.5 g/10 min (at 190 °C, 2.16 kg), and its melting temperature is 47 °C.

The partially disentangled PP was obtained by dissolving 1 wt.% or 0.5 wt.% PP in xylene (Polskie Odczynniki Chemiczne, Warsaw, Poland) at a temperature of 135 °C. The polymer and solvent were mixed in a flask for 1 h. After this time, the temperature of the solution was lowered at a rate of 25 °C/h. When the temperature reached 80 °C, the solution became cloudy, which meant that a gel was formed. After cooling the solution to 40 °C, the gel was removed from the flask and dried. Finally, a white powder with a reduced density of macromolecular entanglement was obtained. The details of the disentanglement procedure are described in a previous publication [76] and in the Supplementary Materials.

An important characteristic of the studied materials is the molecular mass between entanglements, M_e_. For two of the examined materials, we determined it previously using the procedure of Eckstein et al. [5]. The following values of M_e_ were obtained: 9900 g/mol for the equilibrium entangled initial PP and 19,100 g/mol for the PP prepared from 0.5 wt.% solution [18]. Additional measurements, performed using the same method, for PP obtained from a solution with a concentration of 1 wt.%, gave the result M_e_ = 14,960 g/mol. These results confirm the opinion that polymers with less entangled macromolecules are obtained from more dilute solutions.

Blends of differently disentangled PP and equilibrium entangled HDPE, with the addition of 5 wt.% of EOC compatibilizer, were formed using a co-rotating, twin-screw mini-extruder from Zamak-Mercator (Krakow, Poland). The following blending conditions were used: a temperature of 180 °C, a time of 3 min, and a screw rotation of 150 rpm. The list of prepared blends is given in Table 1 (see Section 2). In some experiments, the blends were compared with homopolymers. The PE and PP homopolymers used in these studies were not processed by extrusion.

### 3.2. Methods of Characterization

The rheological properties of the blends were studied using an Ares G2 (TA Instruments, New Castel, DE, USA) rheometer in a plate–plate configuration. Samples with a diameter of 25 mm and a thickness of 1 mm were melted at 175 °C and the frequency sweep test was performed in the frequency range of 0.1–612 rad/s at 1% strain. The loss modulus and storage modulus were determined. Three samples of each material were measured.

The morphologies of the blends were analyzed using a Jeol JSM 6010LA (Jeol, Tokyo, Japan) scanning electron microscope (SEM). Before observations, the blend samples were fractured in liquid nitrogen and the exposed surfaces were sputtered with gold. The average size of the dispersed phase and the number size distribution were determined from photographs taken at 3000× magnification. At least 170, and typically more than 200, dispersed polymer inclusions were measured for each blend.

The changes in the masses of samples during heating to high temperatures and the derivatives of these changes were determined using a TGA 5500 apparatus (TA Instruments, New Castle, DE, USA). The thermogravimetric experiment was carried out in airflow in a temperature range from 25 to 500 °C with a heating rate of 10 °C/min. The mass of the sample was about 4 mg.

The DSC Q-20 differential scanning calorimeter (TA Instruments, New Castle, DE, USA) was used to study the isothermal crystallization. Samples with a total mass of 7–8 mg were placed in aluminum pans and lightly pressed. During the experiment, the blends were melted for 2 min at 190 °C and then the temperature was lowered at a rate of 30 °C/min to the selected PP crystallization temperature: 135 °C or 137 °C. The crystallization process was considered complete when no further changes in heat flow were recorded. The crystallization of PE was observed similarly but at a lower temperature of 123 °C or 125 °C. Typically, the crystallization of PE was studied in the same sample after the crystallization of PP was completed and the temperature was lowered at a rate of 30 °C/min to the desired level. To determine the degree of crystallinity, it was assumed that the crystallization heat of 100% crystalline PP and PE was 209 J/g [77] and 293 J/g [78], respectively. The measurements of the heat of fusion or heat of crystallization for the polymer in the blend took into account the actual mass of the crystallizing component, i.e., its share in the total mass of the sample. In some experiments, to confirm the obtained crystallinity, the samples after isothermal crystallization were melted at a rate of 10 °C/min. DSC measurements were repeated three times.

The crystal growth rates were determined during isothermal crystallization using a Linkam TAHMS 600 (Linkam Scientific Instruments, Redhill, UK) hot stage connected to the Nikon (Nikon Corp., Tokyo, Japan) polarizing light microscope. Preliminary studies showed that in the tested blends, it was possible to determine the crystal growth rate only when the main component was PP. In this case, the growing spherulites were large enough to be measured. It was not possible to determine the crystal growth rate in the dispersed PP or in the PE matrix, because the objects formed were too small. Therefore, measurements of the spherulite growth rate, equivalent to the lamellar crystal growth rate, were only performed for PPe, PP1, PP05, PPe/PE (76:19), PP1/PE (76:19), and PP05/PE (76:19). To perform these measurements, a small amount of the homopolymer or blend was placed between microscope coverslips, melted on a hot stage for 2 min at 190 °C and then pressed to a film of 10–15 µm thickness. This molten sample was cooled at a rate of 10 °C/min to the crystallization temperature of 135 °C. The formation of spherulites in the sample was recorded by a camera connected to a computer. The growth rate was determined based on the position of the spherulite boundary as a function of time. The growth of at least five spherulites was measured in each sample and the results were averaged.

The recorded course of spherulitic structure formation was also used to determine the nucleation density of spherulites. For this purpose, the number of visible spherulites was related to the volume of the observed part of the sample. The observed volume was 0.6 mm × 0.5 mm × 0.01 mm.

Since the growth rate of spherulites depends on the degree of the entanglement of macromolecules, it can be used to evaluate changes in entanglement with the annealing time of a polymer blend. This was shown in the example of the PP05/PE (76:19) blend. The thin film was melted at 190 °C and annealed for different times—2, 17, 32, 47, 62, 77, and 92 min (i.e., in 15 min steps)—and then cooled at a rate of 30 °C/min to the crystallization temperature of 135 °C. At this temperature, the growth rate of PP spherulites was measured. For comparison, similar measurements were performed for the entangled PPe/PE (76:19) blend.

The mechanical properties of the blends were tested in uniaxial tension at 60 °C using an Instron 5582 (Instron, Norwood, MA, USA) universal machine. The choice of elevated temperature was based on the observation that at 25 °C the samples fractured at small strains, and much larger strains could be expected at higher temperatures, with visible differences in the properties of the blends. The strain rate was 10%/min and the dimensions of the narrow part of dog-bone-shaped samples were 12.5 × 5 × 0.5 mm. For each examined material, the results were averaged from 5 measurements. The surface morphologies of the deformed samples were analyzed using SEM microscopy after coating the surfaces with gold.

X-ray scattering, resulting from cavitation in deformed samples, was investigated in a small-angle X-ray scattering (SAXS) experiment. A GeniX Xenocs (Xenocs, Grenoble, France) X-ray source operating at 50 kV and 1 mA was coupled to a Kiessig-type SAXS camera with a length of 1.2 m. The scattered radiation was recorded using a Pilatus 100 K (Dectris, Baden-Daettwil, Switzerland) detector. The acquisition time was 10 min. The scattering patterns were analyzed using Image J ver. 1.54g (National Institutes of Health, Bethesda, MA, USA) software, and then the total intensity values were determined. This made it possible to compare the scattering on different materials and determine whether nanovoids were created in them.

## 4. Conclusions

It has been shown that using melt extrusion, it is possible to produce a blend of two polymers, one of which had partially disentangled macromolecules, without a significant loss of this disentangled state. By using PP with differently disentangled macromolecules and blending it with PE and an EOC compatibilizer, it was possible to obtain blends characterized by good dispersion of the minority component. The differences in the viscosities of the components, related to disentangling, did not significantly affect the average size of the dispersed phase inclusions, which were in the range of 0.35–0.53 µm. The TGA analysis results showed that the blends had a slightly lower temperature (25–30 °C) at which thermal degradation began compared to the component polymers. The temperature range of the degradation process depended on which component, PE or PP, was dominant, but the reduction in entanglements had no effect on thermal stability.

Rheological studies confirmed that the state of the partial disentanglement of macromolecules was preserved after blending. This was visible when the matrix was PP with a lower density of entanglements, as a reduction in the moduli values, compared to the entangled blend. However, it was difficult to observe the impact of PP disentangled on the rheology of the blend when PP was a minority component. The easier movement of less entangled macromolecules inside small inclusions did not affect the behavior of the blend as a whole.

Both PP and PE crystallized under isothermal conditions. In blends where PP was the main component, the difference in the crystallization rate of PP was visible when PP was less entangled. This was mainly due to the increased crystal growth rate. Crystallization in dispersed polymer droplets was suppressed, required a lower temperature (for PP), or resulted in a lower final crystallinity (for PE). The crystallization of PE, when it was the main component of the blend, was not affected by the change in the entanglement of the dispersed PP. Measurements of the crystal growth rate were possible only for PP and only when this polymer was the main component of the blend. Based on the changes in the spherulite size, it was found that in blends, the crystal growth rate was faster when the macromolecules were less entangled. It was 3.43 μm/min for the blend prepared using entangled PP and 4.91 μm/min for the blend prepared using partially disentangled PP05. The presence of inclusions of the second blend component, i.e., PE, slowed down the crystallization process, but to a similar extent for differently entangled blends.

The measurement of the progress of macromolecular re-entangling, based on the spherulite growth rate in melt-annealed samples, showed that even at high temperatures this does not occur quickly. The time measured for the blend (60 min) was shorter than for a similarly treated homopolymer.

The mechanical properties of the blends depended on which component was the dispersed phase and on the degree of the entanglement of the macromolecules. When the yield stress was analyzed, a small increase in the measured stress was observed for the less entangled blends, probably due to better bonding of the components. The ability to achieve a large strain to break did not depend on the degree of the disentanglement of the macromolecules, as long as the dispersed phase was PE. The stretching of the blends with deformable inclusions proceeded without reaching the strain-hardening stage, where differences between blends could be expected. The deformation of blends in which the harder PP was dispersed in the softer PE was different. The elongation at break increased from 108% to 259% when the less entangled PP was used to prepare the blend. In this case, fewer large voids were formed in the sample, most likely due to better bonding of the components, due to easier penetration of macromolecules into the less entangled polymer. Fewer voids formed meant that they did not quickly transform into cracks, destroying the studied sample.

## Figures and Tables

**Table 1:** List of studied polymers and their blends.

Abbreviation	Material	Composition [wt.%]
PPe	PP, initial, equilibrium entangled	-
PP1	PP, partially disentangled, obtained from 1 wt.% xylene solution	-
PP05	PP, partially disentangled, obtained from 0.5 wt.% xylene solution	-
PE	HDPE, equilibrium entangled	-
PPe/PE (76:19)	Blend of PPe, PE, and EOC	76:19:5
PPe/PE (19:76)	Blend of PPe, PE, and EOC	19:76:5
PP1/PE (76:19)	Blend of PP1, PE, and EOC	76:19:5
PP1/PE (19:76)	Blend of PP1, PE, and EOC	19:76:5
PP05/PE (76:19)	Blend of PP05, PE, and EOC	76:19:5
PP05/PE (19:76)	Blend of PP05, PE, and EOC	19:76:5

**Table 2:** Heat of crystallization (H_c_) and crystallinity (Cr) of PP and PE as homopolymers and components of blends, measured during isothermal crystallization at temperatures of 135 °C and 137 °C (PP) and 123 °C and 125 °C (PE).

Sample	Polypropylene				Polyethylene			
	T = 137 °C		T = 135 °C		T = 125 °C		T = 123 °C	
	H_c_ [J/g]	Cr [%]	H_c_ [J/g]	Cr [%]	H_c_ [J/g]	Cr [%]	H_c_ [J/g]	Cr [%]
PE			-	-			187	64
PPe			100	48			-	-
PP1			100	48			-	-
PP05			104	50			-	-
PPe/PE (76:19)	94	45	96	46			115	36
PP1/PE (76:19)	102	49	97	46			84	39
PP05/PE(76:19)	99	47	97	46			63	43
PPe/PE (19:76)	It did not crystallize		It did not crystallize		206	70	210	72
PP1/PE (19:76)	It did not crystallize		It did not crystallize		209	71	209	72
PP05/PE (19:76)	It did not crystallize		It did not crystallize		214	73	208	71

**Table 3:** Growth rate of PP spherulites in homopolymers and blends measured at 135 °C and the results of spherulite nucleation density measurements in films examined by polarizing optical microscopy.

Material	Growth Rate [µm/min]	Density of Nucleation [Nuclei/m^3^]
PPe	3.90	2.01 × 10^13^
PP1	4.83	1.85 × 10^13^
PP0.5	5.40	1.63 × 10^13^
PPe/PE (76:19)	3.43	3.24 × 10^13^
PP1/PE (76:19)	4.41	2.75 × 10^13^
PP05/PE (76:19)	4.91	2.09 × 10^13^

**Table 4:** Tensile properties of the examined blends: σ_y_—yield stress; ε_y_—yield strain; ε_b_—elongation to break.

Material	σ_y_ [MPa]	ε_y_ [%]	ε_b_ [%]
PPe/PE (76:19)	14.8 ± 0.3	19 ± 1	217 ± 91
PP1/PE (76:19)	16.2 ± 0.4	18 ± 1	128 ± 66
PP05/PE (76:19)	15.8 ± 0.3	21 ± 1	173 ± 74
PPe/PE (19:76)	10.6 ± 0.4	25 ± 1	108 ± 62
PP1/PE (19:76)	11.3 ± 0.4	26 ± 1	130 ± 36
PP05/PE (19:76)	11.4 ± 0.3	27 ± 1	259 ± 101

## Data Availability

Dataset available on request from the corresponding author due to internal rules of the institute.

## Supplementary Materials

The following supporting information can be downloaded at: https://www.mdpi.com/article/10.3390/molecules30081786/s1, Methods to confirm the disentanglement degree (Figures S1 and S2); Morphologies of blends formed without the addition of a compatibilizer (Figures S3–S5); Degradation studies by TGA (Figure S6); Complementary studies by DSC (Figures S7–S10).

## References

[1] Berry G.C., Fox T.G. (1968). The viscosity of polymers and their concentrated solutions. Adv. Polym. Sci..

[2] Fetters L.J., Lohse D.J., Richter D., Witten T.A., Zirkel A. (1994). The connection between polymer molecular weight, density, chain dimensions, and melt viscoelastic properties. Macromolecules.

[3] Pawlak A. (2019). The Entanglements of Macromolecules and Their Influence on the Properties of Polymers. Macromol. Chem. Phys..

[4] Wang X.H., Liu R., Wu M., Wang Z., Huang Y. (2009). Effect of chain disentanglement on melt crystallization behavior of isotactic polypropylene. Polymer.

[5] Eckstein A., Suhm J., Friedrich C., Maier R., Sassmannshausen J., Bochmann M., Mulhaupt R. (1998). Determination of Plateau Moduli and Entanglement Molecular Weights of Isotactic, Syndiotactic, and Atactic Polypropylenes Synthesized with Metallocene Catalysts. Macromolecules.

[6] Bu H.S., Gu F.M., Bao L., Chen M. (1998). Influence of entanglements on crystallization of macromolecules. Macromolecules.

[7] Ji G., Ni H., Wang C., Xue G., Liao Y.-T. (1996). Concentration Dependence of Crystalline Poly(ethylene terephthalate) Prepared by Freeze-Extracting Solutions. Macromolecules.

[8] Bastiaansen C.W.M., Meyer H.E.H., Lemstra P.J. (1990). Memory effects in polyethylenes: Influence of processing and crystallization history. Polymer.

[9] Pawlak A., Krajenta J. (2023). Progress in Studies of Disentangled Polymers and Composites. J. Compos. Sci..

[10] Wang Y., Liu M., Chen J., Luo J., Min J., Fu Q., Zhang J. (2020). Efficient disentanglement of polycarbonate melts under complex shear field. Polymer.

[11] Fu J., Wang Y., Shen K., Fu Q., Zhang J. (2019). Insight into Shear-Induced Modification for Improving Processability of Polymers: Effect of Shear Rate on the Evolution of Entanglement State. J. Polym. Sci. B Polym. Phys..

[12] Zhang H., Zhao S., Yu X., Xin Z., Ye C., Li Z., Xia J. (2019). Nascent Particle Sizes and Degrees of Entanglement Are Responsible for the Significant Differences in Impact Strength of Ultrahigh Molecular Weight Polyethylene. J. Polym. Sci. B Polym. Phys..

[13] Pawlak A., Krajenta J., Galeski A. (2018). Cavitation phenomenon and mechanical properties of partially disentangled polypropylene. Polymer.

[14] Romano D., Tops N., Andablo-Reyes E., Ronca S., Rastogi S. (2014). Influence of Polymerization Conditions on Melting Kinetics of Low Entangled UHMWPE and Its Implications on Mechanical Properties. Macromolecules.

[15] Hu H., Chen J., Yang T., Wang P., Min J., Fu Q., Zhang J. (2023). Regulation of Entanglement Networks under Different Shear Fields and Its Effect on the Properties of Poly(L-lactide). Ind. Eng. Chem. Res..

[16] Pawlak A., Krajenta J., Galeski A. (2017). The crystallization of polypropylene with reduced density of entanglements. J. Polym. Sci. Part B Polym. Phys..

[17] Sun H., Zhang Y., Peng F., Cao R., Liu Z., Xu T., Li L. (2025). Influence of Entanglement Density on Polymer Glass Transition Temperature. Macromolecules.

[18] Krajenta J., Safandowska M., Pawlak A. (2019). The re-entangling of macromolecules in polypropylene. Polymer.

[19] Barangizi H., Krajenta J., Pawlak A. (2023). The influence of entanglements of macromolecules on the mechanical and thermal properties of polylactide composites with carbon nanotube. Express Polym. Lett..

[20] Barangizi H., Pawlak A. (2022). Crystallization of partially disentangled polypropylene in nanocomposites with aluminum oxide. Polymer.

[21] Chai S.-C., Xu T.-Y., Cao X., Wang G., Chen Q., Li H.-L. (2019). Ultrasmall Nanoparticles Diluted Chain Entanglement in Polymer Nanocomposites. Chin. J. Polym. Sci..

[22] Luo J., Chen J., Liu M., Min J., Fu Q., Zhang J. (2021). Investigating the Influence of Incorporation of Boron Nitride on the Kinetics of Isotactic Polypropylene Entanglement Recovery. Ind. Eng. Chem. Res..

[23] Xie M., Li M. (2007). Viscosity reduction and disentanglement in ultrahigh molecular weight polyethylene melt: Effect of blending with polypropylene and poly(ethylene glycol). Eur. Polym. J..

[24] Schirmeister C.G., Hees T., Dolynchuk O., Licht E.H., Thurn-Albrecht T., Muelhaupt R. (2021). Digitally Tuned Multidirectional All-Polyethylene Composites via Controlled 1D Nanostructure Formation during Extrusion-Based 3D Printing. ACS Appl. Polym. Mater..

[25] Tao G., Chen Y., Mu J., Zhang L., Ye C., Li W. (2021). Exploring the entangled state and molecular weight of UHMWPE on the microstructure and mechanical properties of HDPE/UHMWPE blends. J. Appl. Polym. Sci..

[26] Tang X., Xing J., Yan X., Ye C., Zhang L., Zhang Y., Shu B., Mu J., Wei L., Wang J. (2022). Metallocene Polyolefins Reinforced by Low-Entanglement UHMWPE through Interfacial Entanglements. Adv. Polym. Technol..

[27] Chen Y., Li W., Zhang L., Ye C., Tao G., Ren C., Jiang B., Wang J., Yang Y. (2023). In Situ Synthesized Self-Reinforced HDPE/UHMWPE Composites with High Content of Less Entangled UHMWPE and High Gradient-Distributed Oriented Structures. ACS Appl. Polym. Mater..

[28] Paul D.R., Bucknall C.B., Paul D.R., Bucknall C.B. (2000). Introduction. Polymer Blends. Volume 1: Formulation.

[29] Bartlett D.W., Barlow J.W., Paul D.R. (1982). Mechanical Properties of Blends Containing HDPE and PP. J. Appl. Polym. Sci..

[30] D’Orazio L., Greco R., Mancarella C., Martuscelli E., Ragosta G., Silvestre C. (1982). Effect of the addition of ethylene-propylene random copolymers on the properties of high-density polyethylene/isotactic polypropylene blends: Part 1—Morphology and impact behavior of molded samples. Polym. Eng. Sci..

[31] Khabbaz H.S., Demets R., Gahleitner M., Duscher B., Stam R., Dimitrova A., Fiorio R., Gijsman P., Ragaert K., Gooneie A. (2024). Rheological insights into the degradation behavior of PP/HDPE blends. Polym. Degrad. Stabil..

[32] Lovinger A.J., Williams M.L. (1980). Tensile Properties and Morphology of Blends of Polyethylene and Polypropylene. J. Appl. Polym. Sci..

[33] Li J., Shanks R.A., Long Y. (2000). Mechanical Properties and Morphology of Polyethylene–Polypropylene Blends with Controlled Thermal History. J. Appl. Polym. Sci..

[34] Elmendorp J.J., Maalcke R.J. (1985). A study on polymer blending microrheology: Part 1. Polym. Eng. Sci..

[35] Lin J.H., Pan Y.J., Liu C.F., Huang C.L., Hsieh C.T., Chen C.K., Lin Z.I., Lou C.W. (2015). Preparation and Compatibility Evaluation of Polypropylene/High Density Polyethylene Polyblends. Materials.

[36] Kock C., Gahleitner M., Schausberger A., Ingolic E. (2013). Polypropylene/Polyethylene Blends as Models for High-Impact Propylene–Ethylene Copolymers, Part 1: Interaction Between Rheology and Morphology. J. Appl. Polym. Sci..

[37] Valenza A., La Mantia F.P., Acierno D. (1984). Rheological characteristic of blends of isotactic polypropylene with high density polyethylene. Eur. Polym. J..

[38] Al-Mulla A., Shaban H. (2014). Study of compatibility of recycled polypropylene/high density polyethylene blends using rheology. Polym. Bull..

[39] Wenig W., Meyer K. (1980). Investigation of the crystallization behavior of polypropylene-polyethylene blends by optical microscopy. Coll. Polym. Sci..

[40] Noel O.F., Carley J.F. (1975). properties of polypropylene-polyethylene blends. Polym. Eng. Sci..

[41] Sherman E.S. (1984). Reinforcement of polyethylene with propylene by a blending and deformation process. J. Mater. Sci..

[42] Varin R.A., Djokovic D. (1988). The effect of annealing at 135^o^ on the mechanical properties of injection molded high density polyethylene-polypropylene blends. Polym. Eng. Sci..

[43] Jose S., Aprem A.S., Francis B., Chandy M.C., Werner P., Alstaedt V., Thomas S. (2004). Phase morphology, crystallization behaviour and mechanical properties of isotactic polypropylene/high density polyethylene blends. Eur. Polym. J..

[44] Jones H., McClements J., Ray D., Hindle C.S., Kalloudis M., Koutsos V. (2023). Thermomechanical Properties of Virgin and Recycled Polypropylene -High-Density Polyethylene Blends. Polymers.

[45] Teh J.W. (1983). Structure and Properties of Polyethylene-Polypropylene Blend. J. Appl. Polym. Sci..

[46] Martuscelli E., Pracella M., Avella M., Greco R., Ragosta G. (1980). Properties of polyethylene-polypropylene blends: Crystallization behavior. Makromol. Chem..

[47] Galeski A., Pracella M., Martuscelli E. (1984). Polypropylene spherulite morphology and growth rate changes in blends with low-density polyethylene. J. Polym. Sci. Polym. Phys. Ed..

[48] Martuscelli E., Pracella M., Della Volpe G., Greco P. (1984). Morphology, crystallization, and thermal behavior of isotactic polypropylene/low density polyethylene blends. Makromol. Chem..

[49] Galeski A., Bartczak Z., Pracella M. (1984). Spherulite nucleation in polypropylene blends with low density polyethylene. Polymer.

[50] Bartczak Z., Galeski A., Pracella M. (1986). Spherulite nucleation in blends of isotactic polypropylene with high-density polyethylene. Polymer.

[51] Eder M., Włochowicz A. (1984). Analysis of the kinetics of crystallization of polyethylene-polypropylene blends. Acta Polym..

[52] Shanks R.A., Li J., Yu L. (2000). Polypropylene-polyethylene blend morphology controlled by time-temperature-miscibility. Polymer.

[53] Rybnikar F. (1988). Crystallization and morphology in blends of isotactic polypropylene and linear polyethylene. J. Macrom. Sci. Part B Phys..

[54] Mehrabi-Mazidi M., Sharifi H. (2021). Post-consumer recycled high-density polyethylene/polypropylene blend with improved overall performance through modification by impact polypropylene copolymer: Morphology, properties and fracture resistance. Polym. Int..

[55] Teh J.W., Rudin A., Keung J.C. (1994). A review of polyethylene-polypropylene blends and their compatibilization. Adv. Polym. Technol..

[56] Chukov N.A., Ligidov M.K., Pakhomov S.I., Mikitaev A.K. (2017). Polypropylene polymer blends. Russ. J. Gen. Chem..

[57] de Ballesteros O.R., Rispo A., Femina G., Davide S., Nocella F., Romano R., Cipullo R., Auriemma F. (2025). Compatibilization of isotactic polypropylene (iPP) and polyethylene (PE) with PP-based olefin block copolymers. Polymer.

[58] Graziano A., Jaffer S., Sain M. (2018). Review on modification strategies of polyethylene/polypropylene immiscible thermoplastic blends for enhancing their mechanical behavior. J. Elastom. Plast..

[59] Vervoort S., den Doedler J., Tocha E., Genoyer J., Walton K.L., Hu Y., Munro J., Jeltsch K. (2018). Compatibilization of Polypropylene-Polyethylene Blends. Polym. Eng. Sci..

[60] Kazemi Y., Kakroodi A.R., Rodrigue D. (2015). Compatibilization Efficiency in Post-Consumer Recycled Polyethylene/Polypropylene Blends: Effect of Contamination. Polym. Eng. Sci..

[61] Tselios C., Bikaris D., Maslis V., Panayiotou C. (1998). In situ compatibilization of polypropylene-polyethylene blends: A thermomechanical and spectroscopic study. Polymer.

[62] Lin Y., Yakovleva V., Chen H., Hiltner A., Baer E. (2009). Comparison of olefin copolymers as compatibilizers for polypropylene and high-density polyethylene. J. Appl. Polym. Sci..

[63] Kruszynski J., Nowicka W., Rozanski A., Liu Y., Parisi D., Yang L., Pasha F.A., Bouyahyi M., Jasinska-Walc L., Duchateau R. (2024). iPP/HDPE blends compatibilized by a polyester: An unconventional concept to valuable products. Sci. Adv..

[64] Klimovica K., Pan S., Lin Y., Peng X., Ellison C.J., LaPointe A.M., Bates F.S., Coates G.W. (2020). Compatibilization of iPP/HDPE Blends with PE-h-iPP Graft Copolymers. ACS Macro Lett..

[65] Lin T.-W., Padilla-Velez O., Kaewdeewong P., LaPointe A.M., Coates G.W., Eagan J.M. (2024). Advances in Nonreactive Polymer Compatibilizers for Commodity Polyolefin Blends. Chem. Rev..

[66] Liao H., Gao J., Liu C., Tao G. (2024). Rheological Investigation on a Polypropylene/Low Density Polyethylene Blending Melt. J. Polym. Mater..

[67] Sangroniz L., Carmeli E., Vulusic L., Hristov V., Galatini M., Tranchida D., Cavallo D. (2025). Lamellar thickness of the polypropylene matrix determines surface induced nucleation of polyethylene droplets in immiscible blends. Polymer.

[68] Namnidi S.D., van Breemen L.C.A., Loojmans S. (2025). Structure-property relations in PP/HDPE blends: From processing to performance. Polymer.

[69] Liu J., Li Y. (2024). Compatibility evaluation and mechanical properties of isotactic polypropylene/high density polyethylene (iPP/HDPE) blends. J. Polym. Res..

[70] Sun Q., Fu Q., Xue G., Chen W. (2001). Crystallization Behavior of Syndiotactic Poly(propylene) Freeze-Dried from Toluene at Very Dilute Concentration. Macromol. Rapid Comm..

[71] Hoffman J.D., Miller R. (1988). Test of the reptation concept: Crystal growth rate as a function of molecular weight in polyethylene crystallized from the melt. Macromolecules.

[72] Hoffman J.D., Miller R. (1997). Kinetic of crystallization from the melt and chain folding in polyethylene fractions revisited: Theory and experiment. Polymer.

[73] Yamazaki S., Hikosaka M., Toda A., Wataoka I., Gu F. (2002). Role of entanglement in nucleation and ‘melt relaxation’ of polyethylene. Polymer.

[74] Haward R.N. (1993). Strain hardening of thermoplastics. Macromolecules.

[75] (2022). Plastics. Determination of the Melt Mass-Flow Rate (MFR) and Melt Volume-Flow Rate (MVR) of Thermoplastics Part 1: Standard Method.

[76] Krajenta J., Pawlak A., Galeski A. (2016). Deformation of Disentangled Polypropylene Crystalline Grains into Nanofibers. J. Polym. Sci. B Polym. Phys..

[77] Krigbaum W.R., Uematsu I. (1965). Heat and entropy of fusion of isotactic polypropylene. J. Polym. Sci.-Part A Gen. Pap..

[78] Wunderlich B., Czornyj G. (1977). A Study of Equilibrium Melting of Polyethylene. Macromolecules.

