# Supplementary material for: Influence of Partial Disentanglement of Macromolecules on the Rheological, Thermal, and Mechanical Properties of Polypropylene–Polyethylene Blends
Source: Molecules. 2025 Apr 16;30(8):1786. doi: 10.3390/molecules30081786 (PMC12029187; doi:10.3390/molecules30081786)
Supplement: Supplementary file 1 [file molecules-30-01786-s001.zip › molecules-3548611-supplementary.pdf]

SUPPLEMENTARY MATERIALS

Influence of partial disentanglement of macromolecules on the rheological, thermal and mechanical properties of polypropylene – polyethylene blends

Justyna Krajenta, Magdalena Lipinska and Andrzej Pawlak

Description of disentangling and confirmation of disentanglement level.

Among the methods leading to a reduction in the density of macromolecular entanglements, the solvent method is most frequently chosen. In dilute solutions, the macromolecular coils move away from each other and become disentangled. The degree of disentanglement can be controlled by the concentration of the solution. To solidify the disentangled polymer, for example, it can be frozen in liquid nitrogen and the solvent removed by sublimation. In the case of PP, which is hot-soluble in xylene, stabilization of the disentangled state can be achieved by partial crystallization during cooling of the solution. At temperature of approximately 80 °C a gel is formed, from which, after removing the solvent, a partially disentangled polymer powder is obtained. In the case of PP, solution concentrations in the range of 0.05-5 wt.% are used. Too little entanglement of macromolecules is not beneficial for the cohesion of the material in the solid state.

Confirmation of partial disentanglement of macromolecules can be obtained by rheological test, mechanical property test (strain hardening phase), or crystallization rate test.

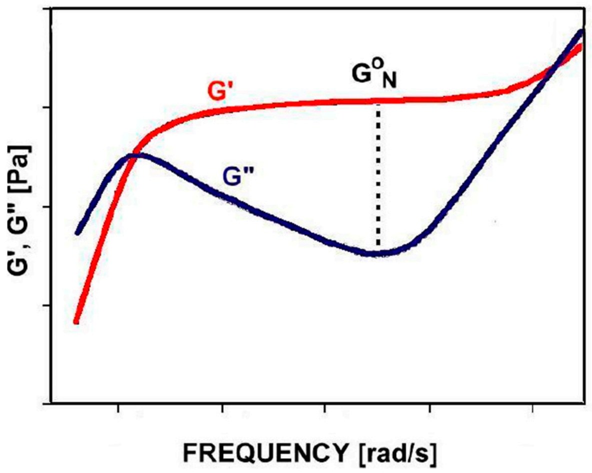

Figure S1. Storage (G’) and loss (G”) moduli for monodisperse long-chain polymer in the function of frequency.

Figure S1 shows the shape of the storage and loss moduli versus frequency during rheological tests in the melt. The modulus of G^o_N in the plateau region is inversely proportional to the molecular mass between entanglements (M_e) and hence M_e can be calculated. Unfortunately, G' and G" values are usually available only for a limited frequency range, as for our PPs shown in Fig. S2. If the maximum G" has been reached in the investigated range, the approach proposed by Eckstein et al. [5] can be used, which involves determining G^o_N from the integral under the loss modulus-frequency curve.

Smaller values of the G' modulus as a function of frequency as in Figure S2 indicate a reduction of macromolecular entanglement.

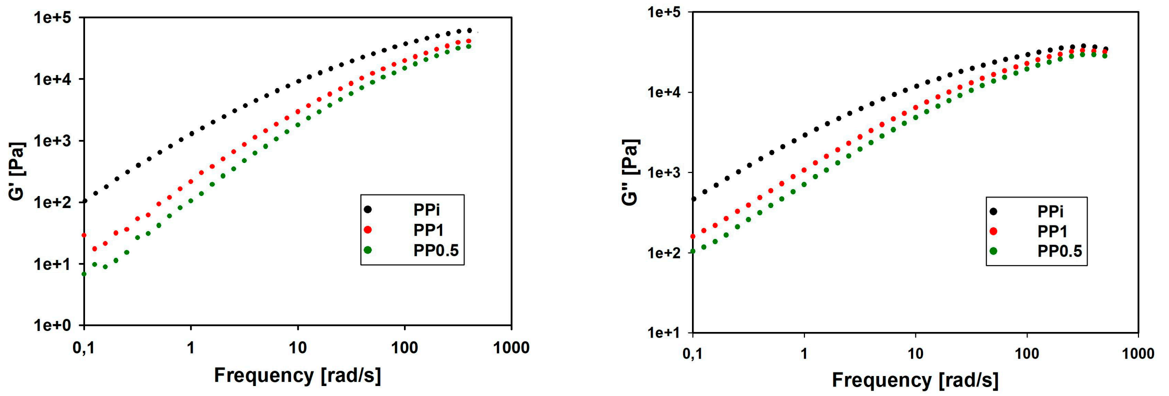

Figure S2. Storage modulus (G’) and loss modulus (G’) as a function of frequency for polypropylenes: fully entangled (PPi), obtained from a 1 wt.% solution in xylene (PP1) and obtained from a 0.5 wt.% solution in xylene (PP0.5). The rheological measurements were performed at 185 °C with strain of 1%.

Morphologies of blends formed without the use of a compatibilizer.

Blends of PP and PE are considered incompatible and are usually characterized by large inclusions of the dispersed phase. As part of the preliminary studies on polymer blends containing disentangled macromolecules, we prepared two blends shown in Figures S3 and S4. We used the same polymers as described in the main publication. Figure S3 shows the morphology of the PPi/PE blend with a weight composition of 80:20, observed using SEM.

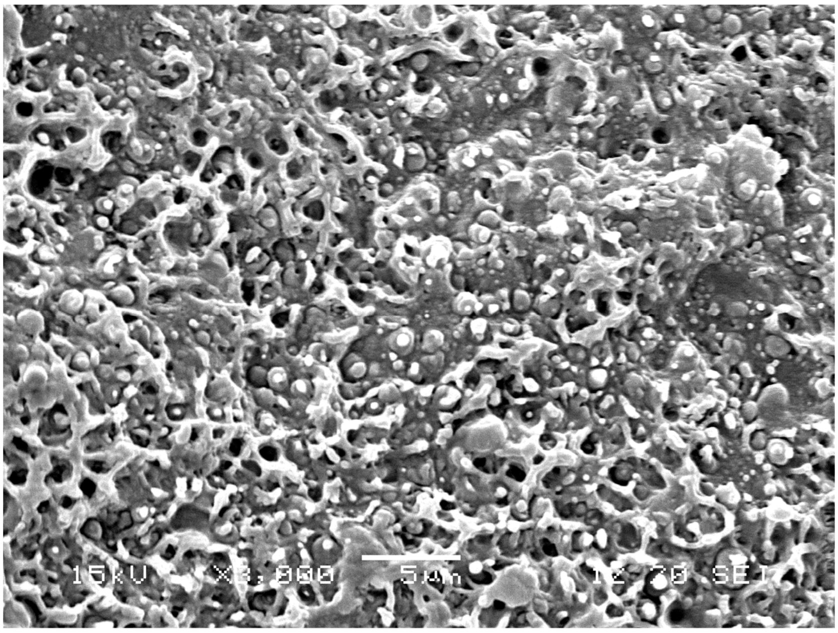

Figure S3. Morphology of PPi and PE blend with a weight composition of 80:20, observed using SEM.

PE inclusions are large, several microns in size. Their separation from the matrix is also visible, indicating poor adhesion of the components.

We checked whether a similar degree of separation of the minority component would occur in a blend made from disentangled polymers. For this purpose, both disentangled polypropylene and

disentangled polyethylene were prepared from a 1 wt.% solution. They were then blended using an extruder. The same proportion of components was used: 80 wt.% PP and 20 wt.% PE. The morphology of the obtained blend is presented in the microscopic photo (Figure S4). Large inclusions and separation of components are visible. Similarly, in the case of the non-compatibilized PP05/PE (20:80) blend shown in Figure S5, there are visible large inclusions of the dispersed phase. With such morphologies, acceptable mechanical properties could not be expected. For this reason, a compatibilizer was used in further studies.

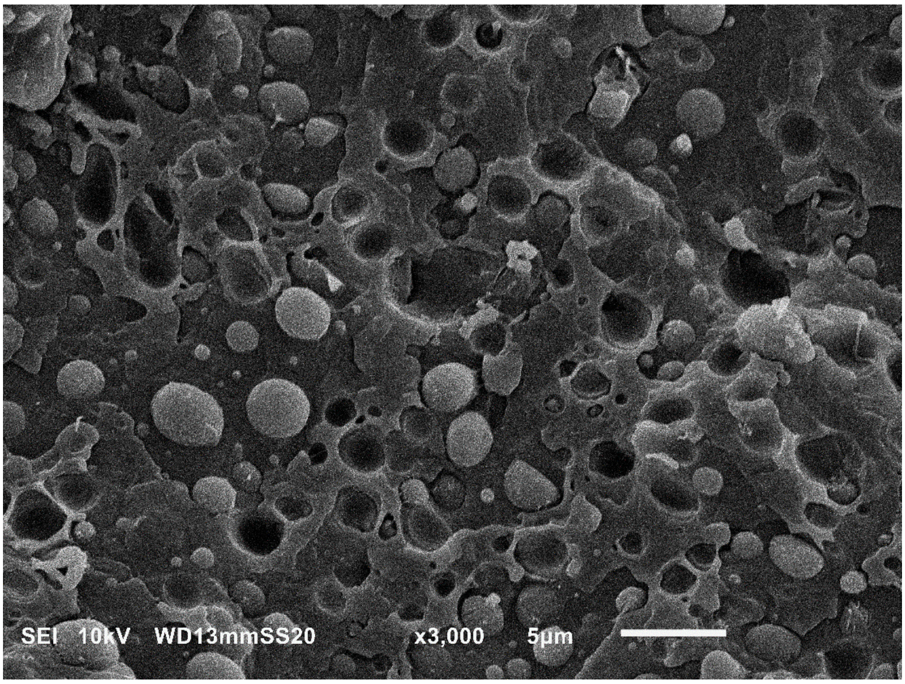

Figure S4. Morphology of the blend prepared form partially disentangled PP and PE (80:20 wt.%).

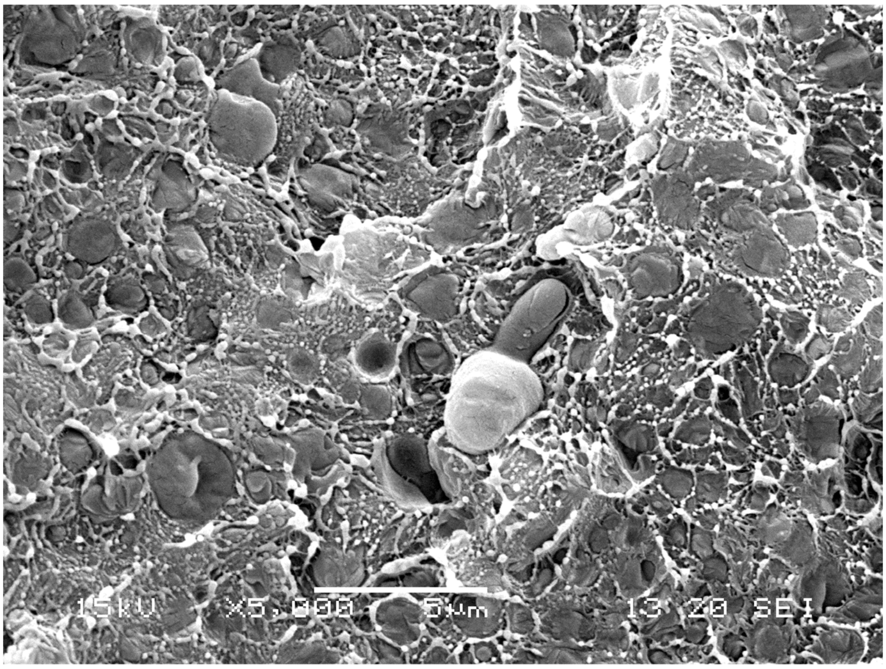

Figure S5. Morphology of the blend PP05/PE (20:80 wt.%).

Degradation studied by TGA.

In some of the studies described in the main text (e.g. crystallization) homopolymers were used that had not been previously processed in the extruder. They were compared with blends that were produced in the extrusion process. Discussing the thermogravimetric results, we showed that mass loss starts at a lower temperature in the blends than in the homopolymers. This may suggest the influence of processing on earlier degradation of the blends. To eliminate these doubts, extrusion of the entangled polymers was performed under the same conditions as in the case of the blends. The TGA results for the PE pellets, PP pellets and the extruded polymers are shown in Figure S6. As can be seen, there is no significant influence of extrusion on the behavior of homopolymers at elevated temperatures.

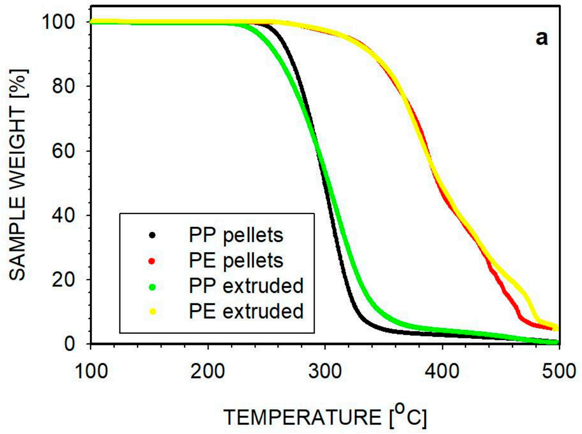

.

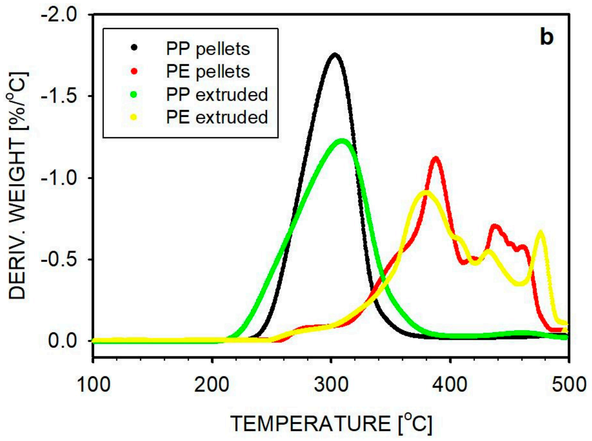

Figure S6. (a) The weight loss during heating of homopolymers in air flow as a function of temperature, (b) The derivative of the weight change of homopolymers as a function of temperature.

Supplementary DSC studies.

Before the isothermal crystallization studies, we performed measurements in a non-isothermal conditions. They showed a good separation of the crystallization temperatures of PP and PE (Figure S7), especially when the crystallization rate was reduced to 2 °C/min (Figure S8).

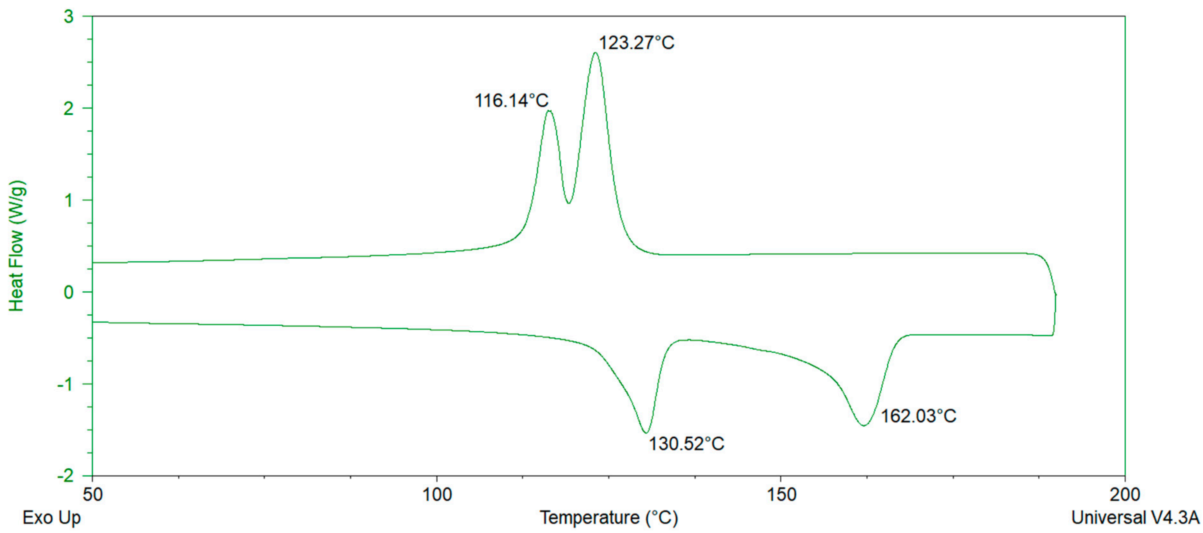

Figure S7. Non-isothermal crystallization of PPe/PE (76:19) performed by DSC, with the rate of heating/cooling 10 ^{o}C/min. Lower curve represents the first heating, upper curve the crystallization.

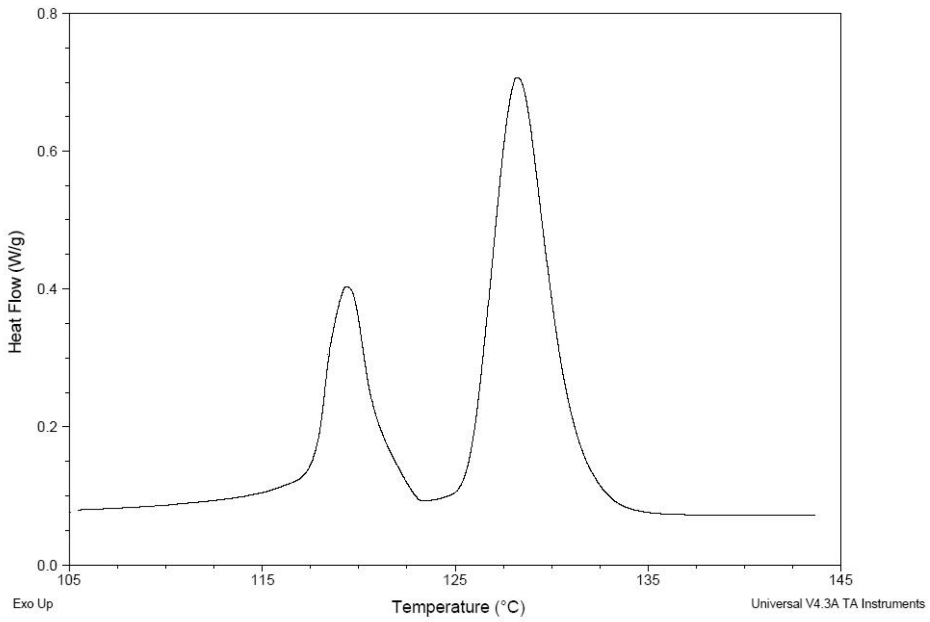

Figure S8. Crystallization of PPe/PE (76:19) during cooling at 2°C/min, measured by DSC.

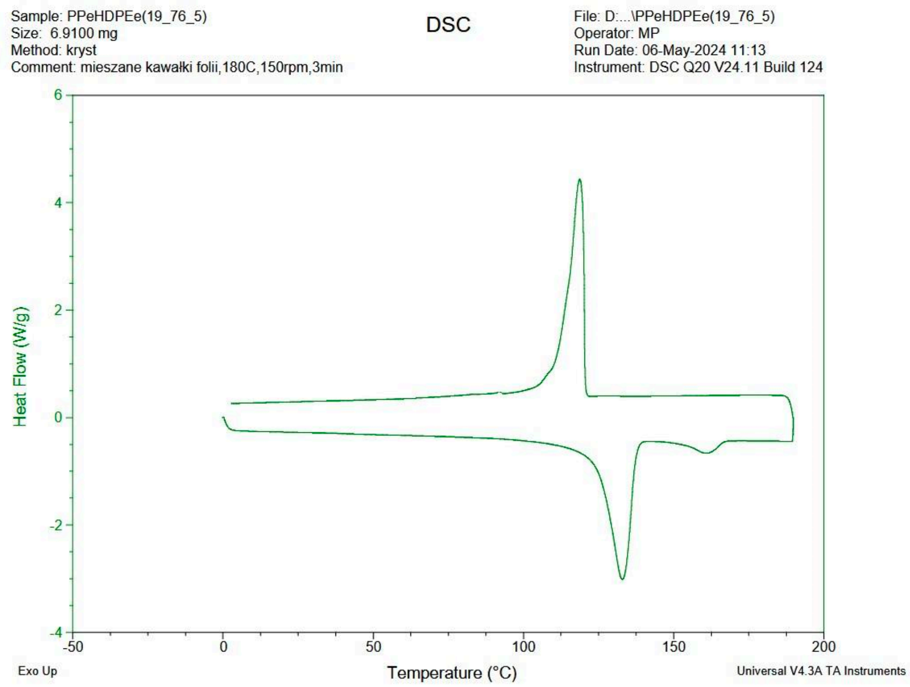

Figure S9. Non-isothermal crystallization of PPe/PE (19:76) performed by DSC with the rate of heating/cooling 10°C/min. Upper curve represents crystallization, lower curve represents second heating.

The crystallization of the PPe/PE (19:76) blend in non-isothermal conditions is shown in Fig. S9. The shape of the crystallization curve indicates that no fractionation crystallization is expected in the PP inclusions during isothermal crystallization.

After performing non-isothermal tests, we established an isothermal crystallization procedure. The crystallization was first carried out at a temperature suitable for PP (133-137 °C), and then the sample was quickly cooled to the temperature suitable for PE crystallization (123-125 °C). An example of a thermograph obtained from DSC device is shown in Figure S10.

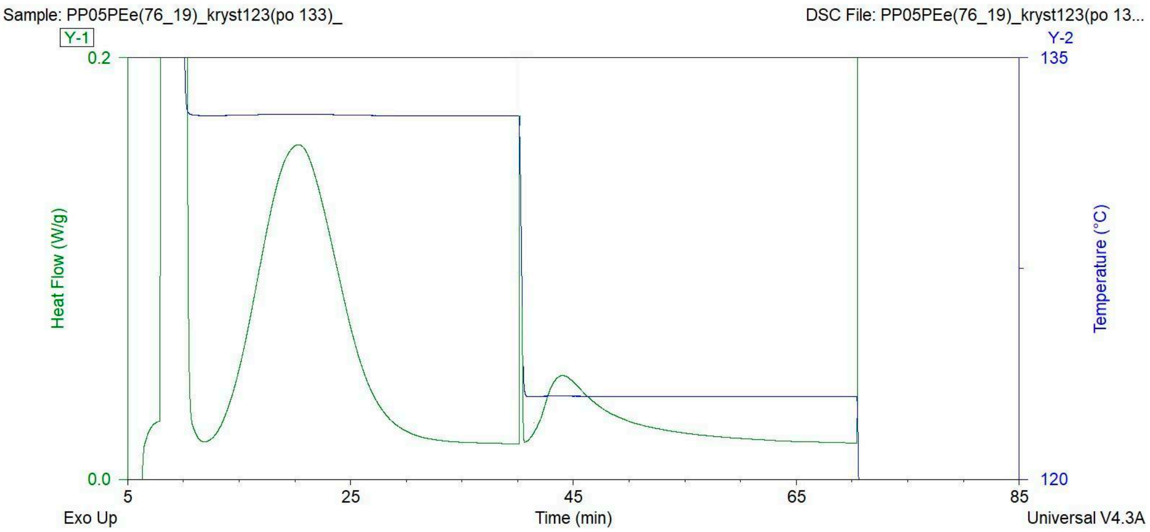

Figure S10. The DSC isothermal crystallization of PP0.5/PE (76:19) showing heat flow and temperature during experiment in which after crystallization of PP05 at 133 °C the crystallization of PE occur after decreasing the temperature to 123 °C.
